# Circa 1 Ga sub-seafloor hydrothermal alteration imprinted on the Horoman peridotite massif

**DOI:** 10.1038/s41598-018-28219-x

**Published:** 2018-06-29

**Authors:** Lalindra V. Ranaweera, Tsutomu Ota, Takuya Moriguti, Ryoji Tanaka, Eizo Nakamura

**Affiliations:** 1 Pheasant Memorial Laboratory for Geochemistry and Cosmochemistry, Institute for Planetary Materials, Okayama University at Misasa, Tottori, 682-0193 Japan; 2 Present Address: Department of Natural Resources, Faculty of Applied Sciences, Sabaragamuwa University of Sri Lanka, Belihuloya, Sri Lanka

## Abstract

The chemical compositions of the residues of the mantle melting that produces mid-ocean ridge basalt can be altered by fluid–rock interactions at spreading ridges and, possibly, during seawater penetration along bending-related faults in plates approaching trenches. This chemically modified rock, if subducted deeply and after long-term residence within the deep Earth, is a potential source of chemical heterogeneity in the mantle. Here, we demonstrate that peridotites from the Horoman massif preserve the chemical signatures of sub-seafloor hydrothermal (SSH) alteration at a mid-ocean ridge approximately one billion years ago. These rocks have evolved chemically subsequent to this SSH alteration; however, they retain the SSH-associated enrichments in fluid mobile elements and H_2_O despite their long-term residence within the mantle. Our results indicate that ancient SSH alteration resulting in the production of sulfide leads to Pb enrichment that could affect the present-day Pb isotopic evolution of the silicate earth. Evidence from the Horoman massif of the recycling of hydrous refractory domains into the mantle suggests that both the flux of H_2_O content into the mantle and the size of the mantle H_2_O reservoir are higher than have been estimated recently.

## Introduction

Mid-ocean ridges (MORs) mark divergent plate margins where new oceanic lithosphere is created, with basalt (mid-ocean ridge basalt; MORB) and complementary peridotite residue (depleted MORB Mantle, DMM^1–3^) resulting from partial melting within the upper mantle. These ridges represent the longest linear features found on Earth’s surface and they contain extensive hydrothermal vent systems^4^. Seawater is able to penetrate downward and rise in temperature through fractured oceanic crust, where it can react with wall rock, before ascending because of its buoyancy and emerging as hydrothermal fluid on the seafloor^4,5^. This hydrothermal fluid can alter the chemical compositions of the oceanic lithosphere, producing enrichments in fluid-mobile elements such as Cs, Rb, U, and Sr^6,7^. Subduction of altered parts of the oceanic lithosphere and the long-term residency of these rocks in the mantle are considered important processes leading to present-day mantle isotopic heterogeneity^8^. Hydration of the lithosphere can lead to the recycling of large amounts of H_2_O into the mantle and to the stabilization of high-pressure hydrous phases such as ringwoodite^9^, affecting mantle rheology and Earth’s seismic structure^10,11^. The presence of altered oceanic crust within the mantle is inferred from the element concentrations and isotopic compositions of igneous rocks representing mantle melts^8^. However, records of hydrothermally altered peridotite in deeply subducted lithosphere have not been identified clearly in the geochemistry of basalts or in exhumed peridotite complexes. The fate of peridotites in subducted lithosphere and their role in the geochemical evolution of the mantle still remain uncertain. In this paper, we describe the Horoman peridotite massif as an example of a hydrothermally altered peridotite representing the residue of MOR melt extraction that was then deeply recycled into the upper mantle and later exhumed as an Alpine-type peridotite massif.

**Figure 1:**
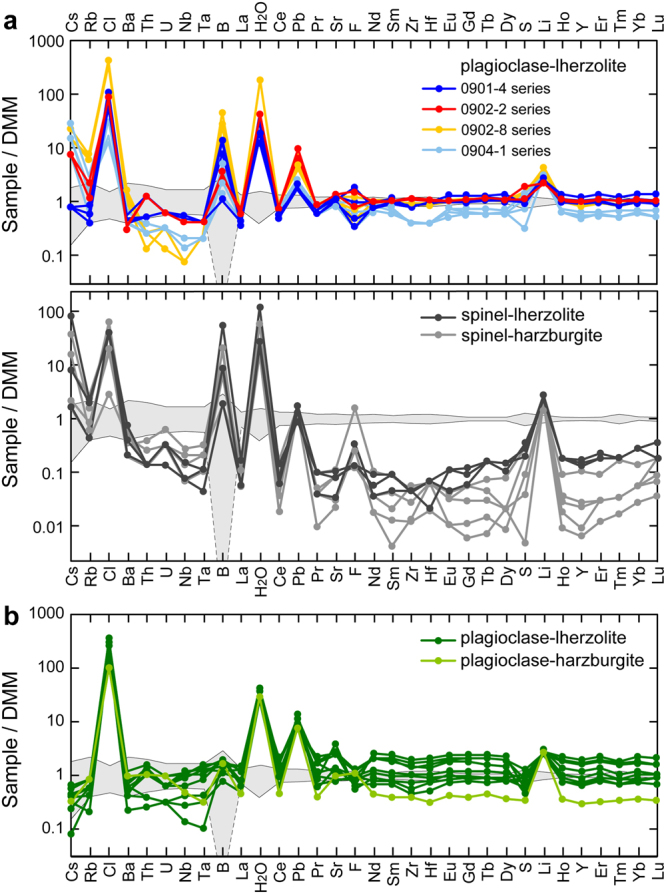
Trace element concentrations of the Horoman peridotites. Element concentrations are normalized to those of depleted MORB mantle, DMM^3,21,65^. Shaded fields show the ranges of element concentrations (±2σ deviations) of the DMM^3,21,65^. The concentrations of B, F, Cl, S, and H (as H_2_O) were determined in this study, but other concentrations were taken from refs^17,18^. Analytical uncertainties (2σ) of elemental abundances are <20%, and the sizes of their corresponding error bars are smaller than those of the symbols. (**a**) Massive peridotite. (**b**) Thin-layer peridotite.

**Figure 2:**
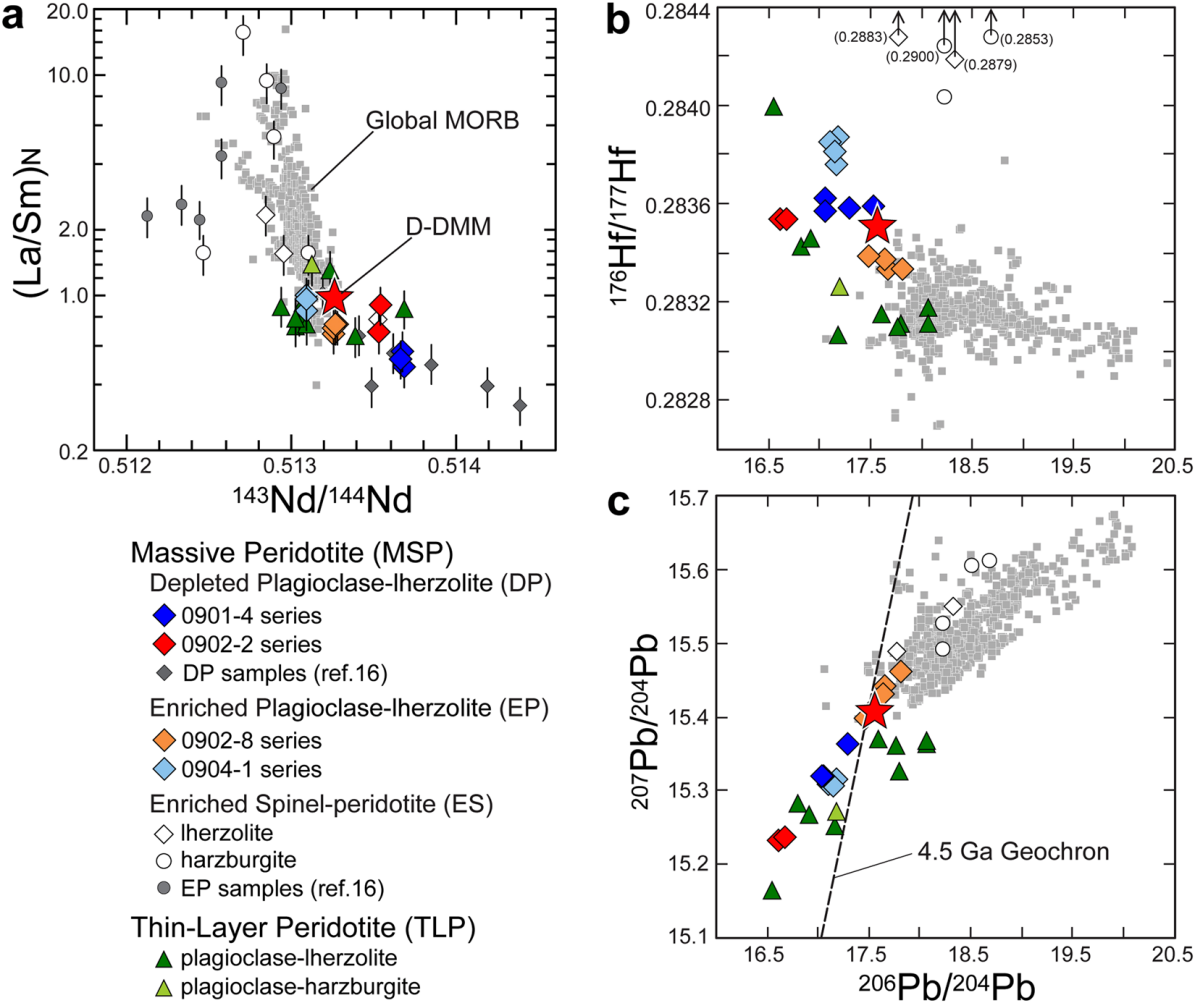
Trace elements and Nd–Hf–Pb isotope compositions of the Horoman peridotites and global MORB. Data for global MORB (gray square) were downloaded from the PetDB Database (http://www.earthchem.org/petdb) on 23 February, 2016. Error bars correspond to 2σ uncertainties (<20%) for elemental abundances^17^. Analytical uncertainties (2σ) of Nd, Hf, and Pb isotope abundances are 0.002, 0.006, and 0.006–0.008%, respectively^17^, and the sizes of their corresponding error bars are smaller than those of the symbols. Data of D-DMM (2σ-depleted, DMM) are from ref.^3^. (**a**) Plot of (La/Sm)_N_ vs ^143^Nd/^144^Nd. Element ratios are normalized to those of the D-DMM. Data of the previous study^16^ are also shown. (**b**) Plot of ^176^Hf/^177^Hf vs ^206^Pb/^204^Pb. (**c**) Plot of ^207^Pb/^204^Pb vs ^206^Pb/^204^Pb.

The Horoman massif, exposed at the southwestern end of the Cenozoic Hidaka metamorphic belt in northern Japan (Supplementary Fig. S1), is a well-preserved Alpine-type peridotite massif containing fertile plagioclase-lherzolite and refractory spinel-lherzolite to highly depleted spinel-harzburgite with subordinate dunite, pyroxenite, and gabbro^12–16^. In terms of their field occurrence, the peridotites of the massif are classified as massive peridotite (MSP) and thin-layer peridotite (TLP); the former occurring as massive rock with thickness >1 m and the latter interlayered with gabbro at millimeter to centimeter scales^17,18^. The MSPs are composed of plagioclase-lherzolite, spinel-lherzolite, and spinel-harzburgite, and the TLPs consist of plagioclase-lherzolite and plagioclase-harzburgite. Both the MSPs and the TLPs contain varying proportions of olivine, clinopyroxene, orthopyroxene, spinel, plagioclase, minor pargasite, sulfide (pentlandite), and serpentine (lizardite, antigorite) (Supplementary Fig. S2). Based on elemental and isotopic characteristics^16–18^, the MSPs can be divided into three types: (1) plagioclase-lherzolite depleted in light rare earth element (REE) abundances and in Nd, Hf, and Pb isotope ratios, compared to their inferred source mantle compositions (Depleted Plagioclase-lherzolite; DP-MSP); (2) plagioclase-lherzolite depleted in light REE abundances but enriched in those isotope ratios (Enriched Plagioclase-lherzolite; EP-MSP); (3) spinel-lherzolite and spinel-harzburgite enriched in light REE abundances and in those isotope ratios (Enriched Spinel-peridotite; ES-MSP) (Figs 1 and 2). The TLP occurrences are more fertile than the MSPs; the former having increased pyroxene modal abundances and enriched incompatible element abundances (Supplementary Table S1) produced by reactions with a MORB-like melt at approximately 300 Ma^18^. Importantly, the TLPs show no obvious evidence of fluid-related processes, and melt-peridotite interactions are not indicated for the MSPs^16–18^.

The Horoman massive plagioclase-lherzolites (P-MSPs) represent residues of polybaric melt extraction from a MORB mantle (DMM) at circa 1 Ga^16,17,19^. Abundances of REEs and the Nd and Hf isotope compositions of the P-MSPs imply these peridotites have existed in closed systems since the extraction of the MOR melt at circa 1 Ga^17^. The DP-MSPs have very low ^206^Pb/^204^Pb, ^207^Pb/^204^Pb, and ^208^Pb/^204^Pb ratios, which are the most unradiogenic Pb isotope values detected in any mantle material, and are regarded as a potential solution for the terrestrial Pb isotope paradox^17,20^. The unradiogenic Pb isotope ratios in the DP-MSPs are consistent with partial melting at circa 1 Ga and subsequent radiogenic evolution, whereas the enrichment of elemental Pb in those peridotites apparently does not reflect such a process^16^. The melting at circa 1 Ga would have depleted incompatible elements, including Pb, in the melt residue. These peridotites are also enriched in elements such as Rb and B, which are considered relatively fluid-mobile^18^. Such isotopic and elemental characteristics cannot have been caused solely by ancient partial melting of the MORB mantle. The objectives of this study were to unravel the peculiar evolution of the Horoman peridotites and to examine the geodynamic implications. To this end, we investigated the concentrations of H, B, Cl, F, and S, isotopic compositions of H, Li, O, and Sr, and modal abundances of the major and trace minerals of MSP and TLP samples, which were examined in previous studies^17,18^ for their major and trace element compositions and Nd–Hf–Pb isotope systematics (Supplementary Tables S1, S2 and S3).

## Results

### Fluid mobile and volatile elements in the Horoman peridotites

All the peridotites examined in this study have H_2_O contents one to two orders of magnitude higher than that of the upper mantle (approximately 0.02 wt.% for the latter; ref.^11^; see Supplementary Table S2). For the P-MSPs, positive anomalies in the concentrations of fluid-mobile elements (Cs, Rb, Li, and Pb) and volatile elements (B and Cl; Fig. 1), relative to the DMM^3,21^, suggest aqueous fluid metasomatism of the residual peridotites. The Cl abundances of the P-MSPs are positively correlated with the abundances of H_2_O and B, extending to the ranges for seawater, hydrothermal vent fluids, serpentinized abyssal peridotite, and arc magmas (Supplementary Fig. S3). Abyssal serpentinites and mantle wedge serpentinites are characterized by the development of U-shaped REE patterns with positive Eu anomalies^7^. In contrast, all of the P-MSPs present smooth, light-REE-depleted patterns that are typical of partial melting residues^17^. Thus, fluid metasomatism has enriched these rocks with the fluid-mobile and volatile elements without noticeable alteration of their MORB-peridotite-like REE abundances.

Although the Horoman peridotites contain pargasite as one of hydrous minerals, the modal abundances of pargasite and the concentrations of H_2_O and fluid-mobile elements show no obvious correlation (Supplementary Fig. S4). Meanwhile, even under X-ray mapping with pixel spacing of 15–20 µm, little serpentine was observed in most of the P-MSPs (Supplementary Table S1). Therefore, the enrichment of the fluid-mobile and volatile elements can be predominantly attributed to another fluid-related process, distinct from the later-stage event that stabilized the pargasite and serpentine presently observed in these rocks.

**Figure 3:**
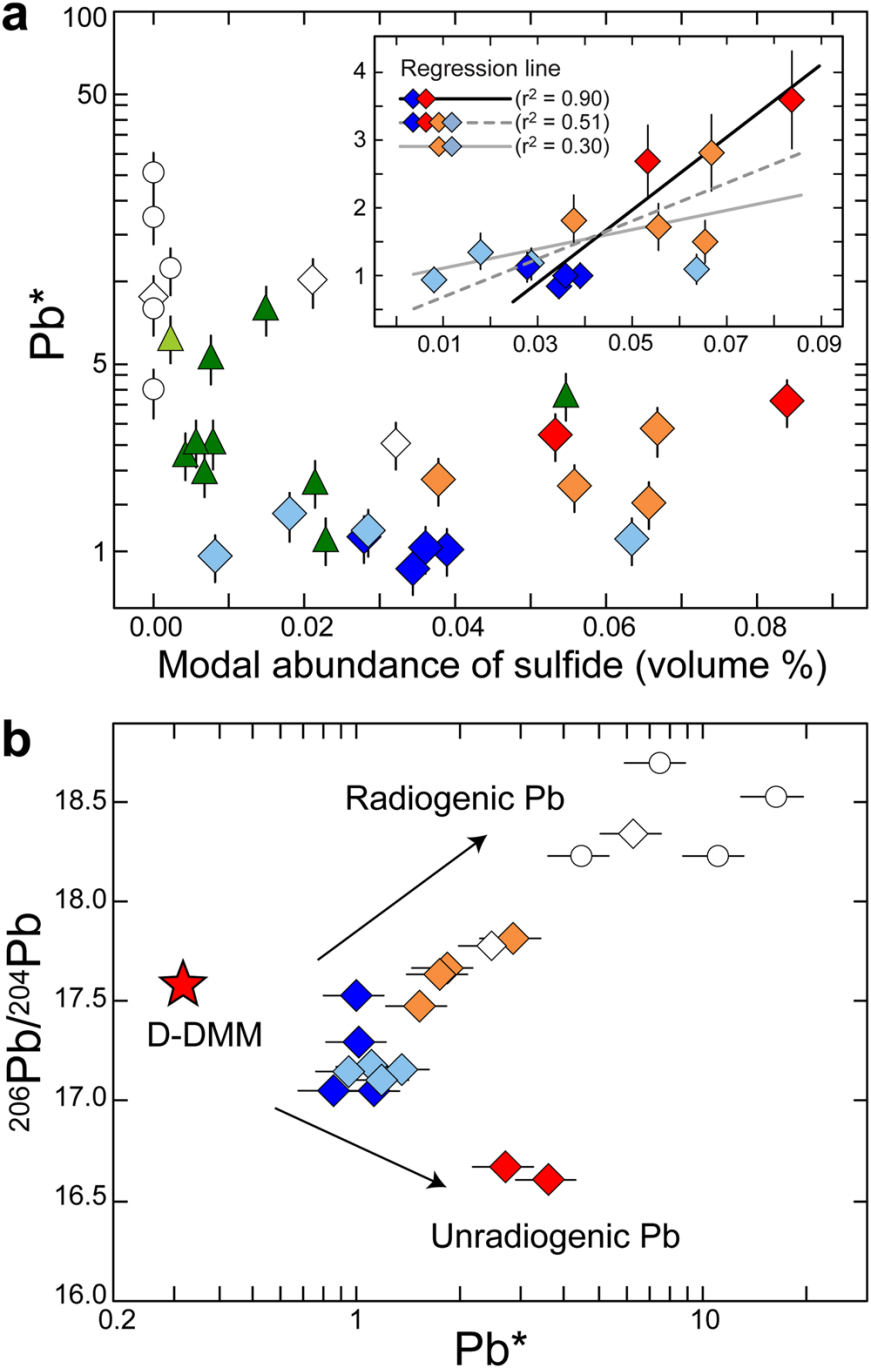
Excess Pb for the Horoman peridotites. Excess Pb is defined as Pb* = [2·Pb/(Ce + Pr)]_N_, where element concentrations are normalized to those of the primitive mantle^22^. Symbols are the same as in Fig. 2. For element concentrations, error bars correspond to 2σ uncertainties (<20%)^17^. Lead isotope compositions are from ref.^17^; reproducibility for their analysis is 0.008% (2σ), and the error bars are smaller than the symbols. (**a**) Correlation with modal abundances of sulfide. The inset is for massive plagioclase-lherzolites. (**b**) Correlation with Pb isotope compositions. Data of D-DMM are from ref.^3^, with calculation after ref.^66^.

Interestingly, the modal abundance of sulfide in the DP-MSPs correlates with excess Pb, defined as Pb* = 2·Pb/(Ce + Pr), where abundances of the elements are normalized to those of the primitive mantle^22^ (Fig. 3a). Sulfides occur in the peridotites through interactions of peridotites with hydrothermal fluid or melt at MORs^5,23^, and the Pb of chalcophile is sequestrated into the sulfide^24^. In the Horoman P-MSPs, most sulfides are anhedral in shape, and their filling grain boundaries of the surrounding minerals (Supplementary Fig. S2) imply that they are secondary products caused by fluid or melt interaction. As the MSPs do not show obvious elemental signatures of melt metasomatism^17,18^, the sulfide abundance and Pb enrichment in the DP-MSPs appear to reflect interactions with hydrothermal fluid at MORs.

### Strontium and lead isotopes in the Horoman MSPs

**Figure 4:**
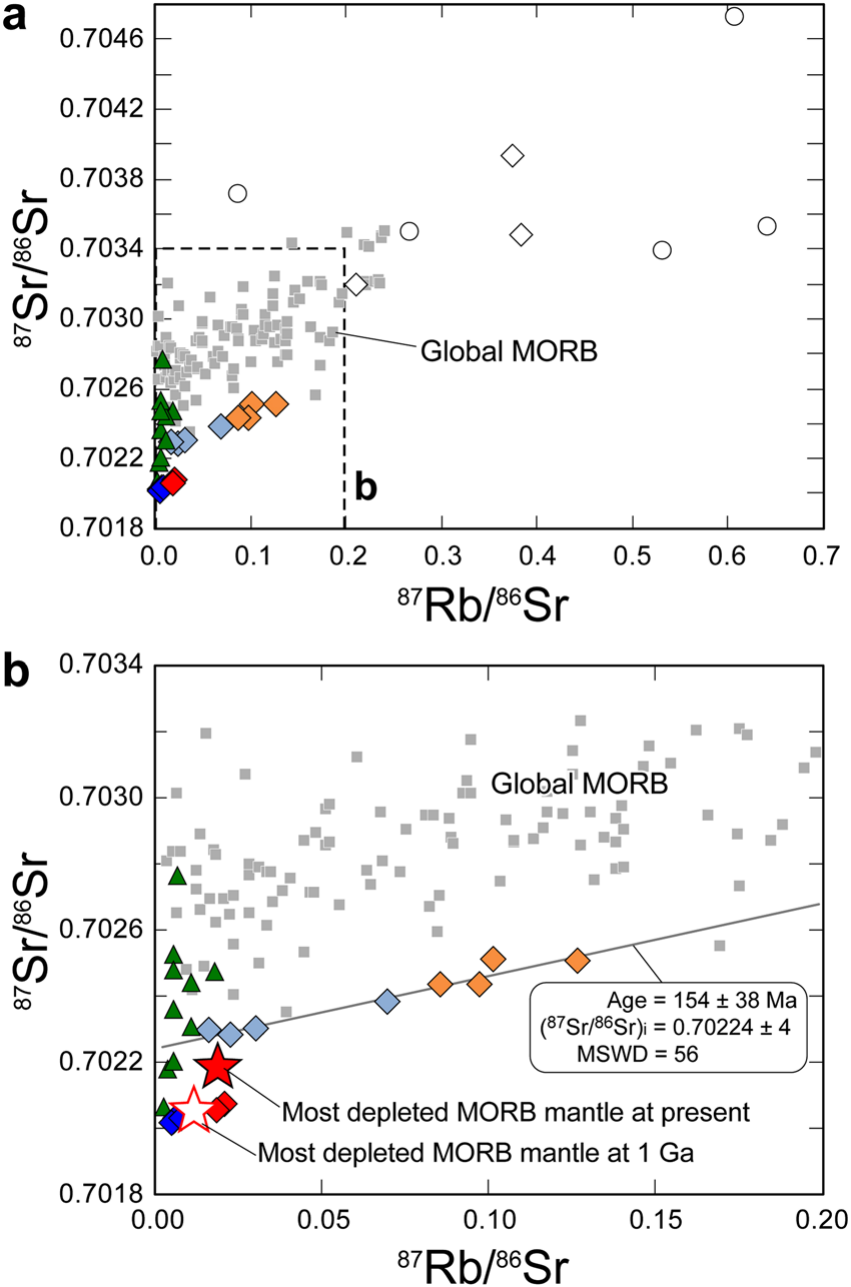
Rubidium and Sr isotope compositions of the Horoman peridotites and global MORB. The data for global MORB (gray square) were downloaded from the PetDB Database (http://www.earthchem.org/petdb) on 23 February, 2016. Symbols are the same as in Fig. 2. Analytical uncertainties (2σ) for the Horoman peridotites are <2% and 0.002% for elemental and isotope ratios, respectively, and the corresponding error bars are smaller than the symbols. (**a**) The entire range of isotope compositions, with the region expanded in (**b**) indicated by the dashed lines. (**b**) Expanded view of the region in (**a**) indicated by the dashed lines. Solid and open stars indicate the compositions of the most depleted MORB mantle at present^3^ and at 1 Ga, respectively.

**Figure 5:**
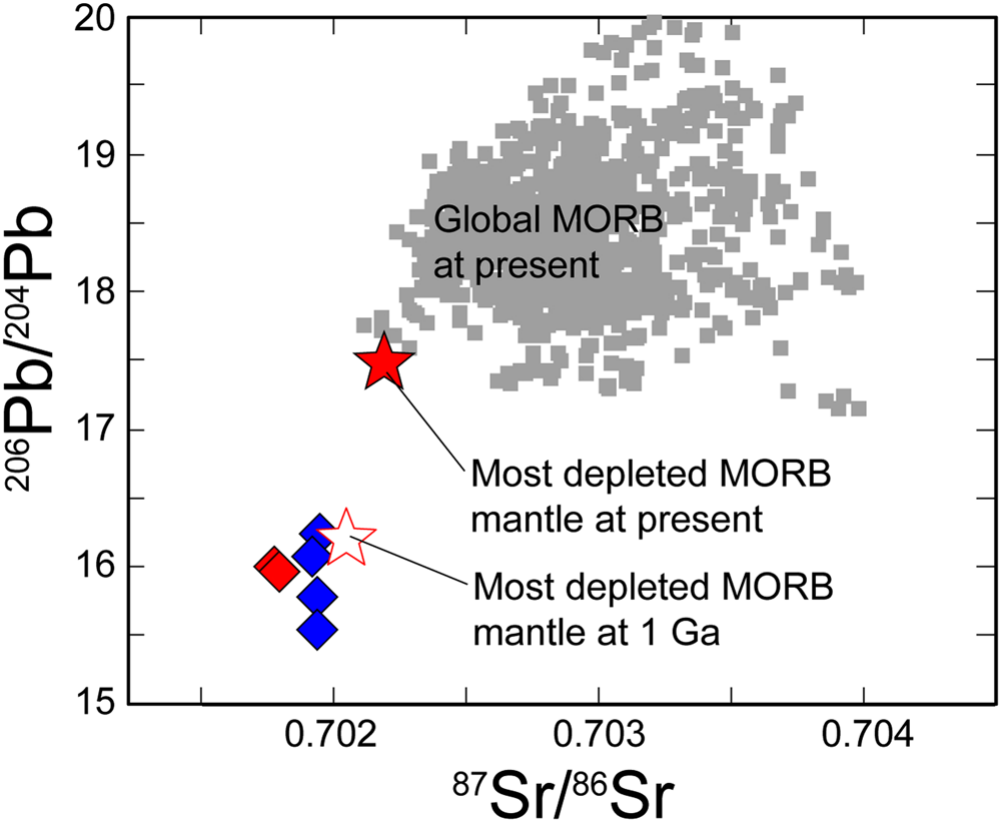
Calculated 1-Ga Sr and Pb isotope compositions for the Horoman peridotites. Lead isotope compositions are from ref.^17^. Analytical reproducibilities (2σ) are 0.002% for ^87^Sr/^86^Sr and 0.008% for ^206^Pb/^204^Pb, and their corresponding error bars are smaller than the symbols. Solid and open stars indicate the isotope ratios of the most depleted MORB mantle at present^3^ and at 1 Ga, respectively. Data for global MORB were downloaded from the PetDB Database (http://www.earthchem.org/petdb) on 23 February, 2016.

The Rb–Sr isotope system of the EP-MSPs was modified at approximately 150 Ma or more recently (Fig. 4). Furthermore, the 0902–8 series samples are enriched in radiogenic Pb (Fig. 3b), implying that their geochemical affinity with the ES-MSPs reflects metasomatism by fluids generated during subduction in the slab^17,18^. On the other hand, the Sr isotope ratios of the DP-MSPs are more depleted than those of the most depleted MORB mantle (Fig. 4); the ^87^Rb/^86^Sr ratio of the 0901–4 series samples is the lowest among the MSPs, indicating that these rocks accumulated little radiogenic Sr over geological time and thus, they best reflect the initial ^87^Sr/^86^Sr ratio of the DP-MSPs, which nearly coincides with that of the most depleted MORB mantle at 1 Ga. The ^87^Sr/^86^Sr ratios of the 0902–2 series samples, which have the highest Pb* values with the most unradiogenic Pb-isotope ratios (Fig. 3b and Supplementary Table S2), are also comparable with the estimated initial value (Fig. 4). Indeed, calculated 1-Ga Sr–Pb isotope ratios of the DP-MSPs are comparable with those of the most depleted MORB mantle at 1 Ga (Fig. 5).

### Oxygen, lithium, and hydrogen isotopes in the Horoman peridotites

**Figure 6:**
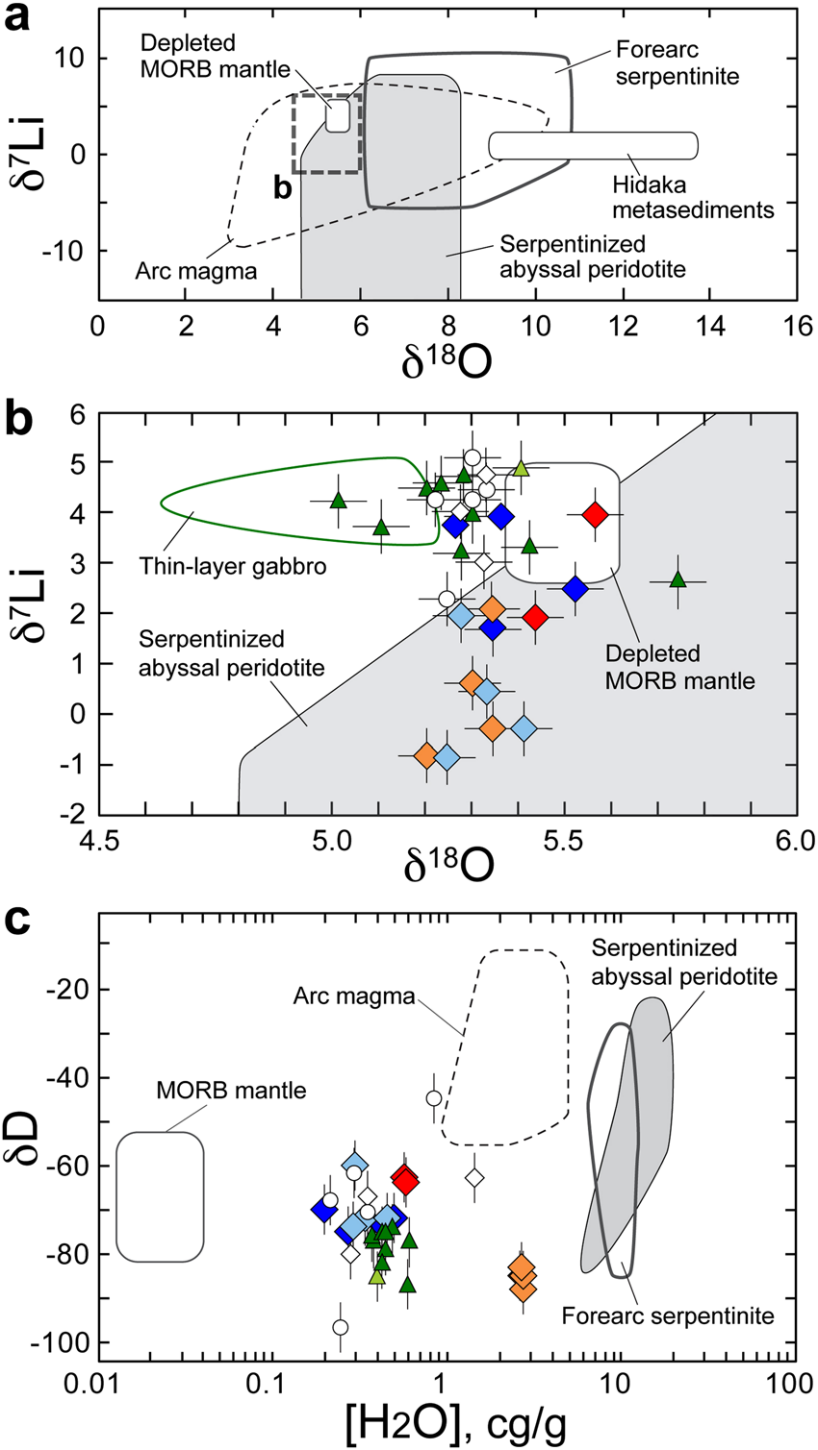
Oxygen, Li, and H isotope compositions, and H_2_O concentrations of the Horoman samples. Symbols are the same as in Fig. 2, except data for arc magma^67,68^, depleted MORB mantle^69,70^, MORB mantle^21,71,72^, serpentinized abyssal peridotite^25,28,73,74^, and forearc serpentinite^26,27,29^. Error bars for isotope compositions correspond to analytical reproducibilities (2σ); those for H_2_O concentration (3.2%, 2σ) are smaller than the symbols. (**a**) Oxygen and Li isotope compositions. The range of the plot displayed in (**b**) is shown by the dashed line. (**b**) Oxygen and Li isotope compositions of the Horoman samples. (**c**) Hydrogen isotope compositions and H_2_O concentrations of the Horoman peridotites.

The δ^18^O of Alpine-type peridotites is often modified during exhumation-related metasomatism; however, the O-isotope compositions of the Horoman peridotites appear largely unmodified by fluids derived from recently juxtaposed Hidaka metasedimentary wall rocks with distinctively high δ^18^O (Fig. 6a). During sub-seafloor hydrothermal (SSH) alteration of abyssal peridotites, δ^18^O values are elevated by low-temperature serpentinization, and forearc serpentinites tend to have higher δ^18^O values compared with serpentinized abyssal peridotites^25–27^ (Fig. 6a). However, the δ^18^O values of the Horoman MSPs are not obviously distinguishable from those of the MORB mantle, but instead they overlap the range for serpentinized abyssal peridotites (Fig. 6b).

The δ^7^Li values of both the serpentinized peridotites formed by SSH alteration (−28‰ to +7‰^28^) and the forearc serpentinites (−6‰ to +10‰^29^) are comparable with or lower than those for the DMM, indicating that serpentinization can modify the Li isotope compositions to become more depleted in ^7^Li than the DMM. The δ^7^Li values of P-MSPs vary significantly (by approximately 5‰). Their range extends to values lower than that of the DMM (Fig. 6b), not only for the EP-MSPs that show recent modification in their Rb–Sr isotope systematics, but also for the DP-MSPs that barely show this recent modification. Experimental studies of Li isotope fractionation in isotopically equilibrated systems have demonstrated that ^6^Li prefers [6]-fold coordinated sites in most ferromagnesian minerals such as clinopyroxene, olivine, and serpentine, i.e., δ^7^Li_mineral_ ≤ δ^7^Li_fluid_^30–33^. Particularly for olivine, which is the most abundant mineral in peridotites, we confirmed this Li isotope fractionation through experiments under upper-mantle conditions (Supplementary Experiment and Fig. S5). Thus, the Li isotope variation in the Horoman MSPs can be attributed to isotope fractionation in equilibrated silicate–fluid systems.

The δD values of serpentinized peridotites at modern MORs^25^ and at the Mariana forearc^26,27^ are comparable with or higher than those of the MORB mantle (Fig. 6c). These values reflect the H isotope composition of modern seawater, low temperatures of seafloor alteration, and high fluid–rock ratios during serpentinization^27^. Although the δD values of the Horoman MSPs overlap those of the modern MORB mantle, the H_2_O concentrations of the Horoman peridotites are far higher than those of mantle rocks (Fig. 6c), consistent with the lack of H isotope equilibration of the MSPs with the mantle.

## Discussion

### Evidence for ancient SSH alteration at a mid-ocean ridge

The Cl abundances of the P-MSPs are positively correlated with the abundances of H_2_O and B, extending to the ranges for seawater, hydrothermal vent fluids, serpentinized abyssal peridotite, and arc magmas (Supplementary Fig. S3). Such correlations suggest that the P-MSPs experienced either SSH alteration at an MOR or slab-derived fluid metasomatism at a subduction zone, or both. Oceanic lithosphere that has experienced SSH alteration at an MOR can again be infiltrated by seawater just prior to subduction during bending-related extensional faulting outward of a trench^34^. The incoming lithosphere is, in most cases, overlain by pelagic and trench-fill sediments having enriched Sr isotope and radiogenic Pb isotope signatures^8^. Thus, the seawater infiltrating the lithosphere could convey these isotopic signatures to sub-crustal depths and into the peridotite. The EP-MSPs could have been altered in terms of their Sr-Pb isotopes (Fig. 4b) during near-trench plate bending. However, the depleted Sr isotope and unradiogenic Pb isotope signatures preserved in the DP-MSPs must reflect ancient SSH alteration at an MOR where there would have been little geochemical influence by terrigenous sediments. The calculated 1-Ga Sr–Pb isotope compositions of the DP-MSPs are comparable with or more depleted than those of the most depleted MORB mantle at 1 Ga (Fig. 5), indicating that the SSH alteration occurred at least circa 1 Ga. While the sulfide abundance–Pb* correlation, which is expected in peridotites containing SSH sulfides, is observed in the DP-MSPs, the Pb* values of the EP-MSPs are poorly correlated with their sulfide abundances (Fig. 3a). This is likely because radiogenic Pb enrichment by fluid activities in near-trench or subduction zone settings does not always involve sulfide production; instead, the radiogenic Pb would be stored in silicate minerlas^35^, as shown by the high Pb* values of the ES-MSPs (Fig. 3a).

As for the stable isotope compositions examined in this study (Fig. 6), the O isotope compositions of the Horoman MSPs are almost the same as those of the MORB mantle; they overlap the range for serpentinized abyssal peridotites, but remarkably distinguished from severely altered forearc serpentinites. Serpentinization can modify the Li isotope compositions to become depleted in ^7^Li compared with the DMM, as supported by experiments on serpentine–fluid Li isotopic fractionation^32^. As serpentine breaks down to release aqueous fluid during the recycling of serpentinized peridotites into the mantle^36^, the resulting olivine would show further lowering in δ^7^Li^30,33^, as confirmed by our experiments under upper-mantle conditions (Supplementary Experiment). It thus appears that the Li isotope variation in the Horoman MSPs, all with δ^7^Li lower than DMM, was facilitated by isotope fractionation in equilibrated silicate–fluid systems during the ancient SSH alteration followed by recycling in the mantle. The Horoman MSPs are highly enriched in H_2_O with δD values overlapping those of the modern MORB mantle; unless the Horoman MSPs have absorbed H_2_O with the low δD value similar to that of the mantle, the mechanism inferred from the Li isotope compositions must have lowered the δD values of the peridotites serpentinized by SSH alteration. When the serpentinized peridotite in oceanic lithosphere subducts into the deep mantle, it transforms through a series of dehydration reactions into less-hydrous ultramafic rocks, in which the H isotope compositions can also become highly fractionated, as shown by the chlorite-harzburgite dehydration products of antigorite serpentinites that have δD values of −94‰ to −55‰^37^. It is likely that the δD values of the Horoman MSPs reflect a shift during the dehydration reactions from values similar to those of the serpentinized peridotites produced during the ancient SSH alteration. In summary, the stable isotope compositions of the Horoman peridotites are not inconsistent with the idea that the Horoman massif includes SSH-altered peridotites which originate from the residues of MOR melt extraction.

**Figure 7:**
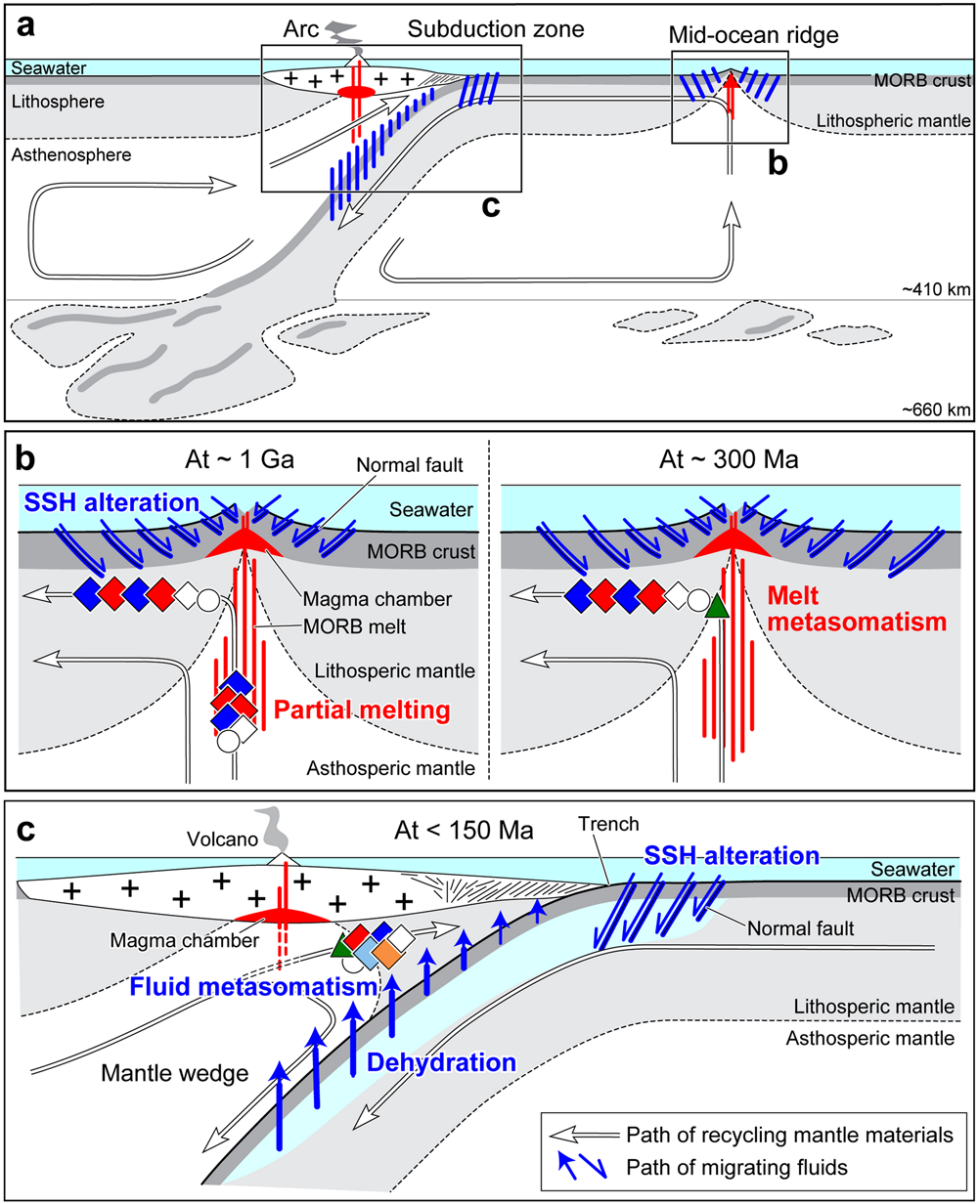
Schematic cross-sections illustrating the evolution of the Horoman massif at different spatial scales of (**a**) upper mantle, (**b**) mid-ocean ridge, and (**c**) subduction zone. Symbols are the same as in Fig. 2. The Horoman peridotites were formed as residues of partial melting of a MORB source at circa 1 Ga^16,17,19^, and underwent the SSH alteration to cause the highly unradiogenic Pb isotope compositions at the present day^17^. The peridotites became part of lithospheric mantle, and recycled back into the mantle. In the peridotites, the H and Li isotopes were fractionated through the SSH alteration and subsequent dehydration under high pressure during recycling in the mantle. With re-processing at different ridge systems, the peridotites interacted with partial melts of MORB mantle at circa 300 Ma^18^. Since circa 150 Ma, the peridotites moved into the mantle wedge setting of the Hidaka subduction zone, and were metasomatized by a slab-derived fluid enriched in radiogenic Sr–Pb isotopes. Prior to unroofing at the surface, the Horoman massif was exhumed to crustal level, juxtaposed with the Hidaka metamorphic belt^75^, and locally metasomatized by a slab-derived fluid at approximately 23 Ma^58^.

Our results suggest that, in the Horoman MSPs, the concentrations of fluid-mobile and volatile elements and the H, Li, O, Sr, Nd, Hf, and Pb isotope compositions were produced by melt extraction from MORB mantle at circa 1 Ga, and the following processes: (i) SSH alteration at the MOR, (ii) dehydration reactions in the oceanic lithosphere during subduction into the mantle, and (iii) recent interaction with subduction-zone fluids derived from the dehydration of the subducting lithosphere that had previously experienced SSH alteration. The ES-MSPs and the EP-MSPs were highly and moderately altered by interaction with subduction-zone fluids in (iii), respectively, because these rocks possess a high fluid permeability related to their olivine-rich matrix^16^, and occur at the periphery of the massif (Supplementary Fig. S1). However, the DP-MSPs were altered least by the subduction-zone fluids in (iii), and it best preserves the initial SSH alteration in (i). To illustrate these processes of the MSPs, with the formation process of the TLPs, we present a model for evolution of the Horoman massif (Fig. 7).

### Potential abundance of SSH altered peridotites in the mantle

In peridotites that experience SSH alteration and then remain closed systems during subduction-zone metamorphism, phases such as serpentine give way to higher-pressure, higher-temperature mineral phases, without affecting whole-rock compositions^36^. During subduction into, and residency within, the upper mantle, sulfides in the peridotites probably also transform into high-pressure phases^38^; however, the DP-MSPs appear to have remained closed chemical systems, at a mineral scale, since the SSH alteration at circa 1 Ga. This closed-system behavior is particularly evident in the correlation between the sulfide abundance and the Pb* value of the DP-MSPs (Fig. 3a). During subduction-zone metamorphism, H_2_O and fluid-mobile elements enriched in hydrous phases during SSH alteration could have been re-distributed into high-pressure silicate phases^10^ despite partial release during dehydration reactions. During the exhumation of the Horoman massif, the H_2_O and fluid-mobile elements could then have been re-distributed into low-pressure hydrous phases where they now reside.

The Horoman massif consists of fertile and refractory mantle segments that experienced melt extraction corresponding to about 10–25% melting of the DMM source^15,16^. The more refractory rocks would have had little ability to produce additional melt^39^. Mid-Atlantic abyssal peridotites contain both interstitial sulfides with relatively radiogenic Pb isotope compositions and sulfide inclusions preserving extremely unradiogenic Pb isotope compositions, the latter yielding ^207^Pb–^206^Pb ages of approximately 1.8 Ga^40^. Sulfides with relatively radiogenic compositions were likely influenced by recent recrystallization, whereas the sulfide inclusions with unradiogenic compositions could have been shielded by host silicates, residing in refractory mantle segments during the melting beneath a spreading ridge. Refractory segments strongly enriched in H_2_O potentially produce harzburgite-saturated, hydrous melt, as shown by melting experiments in a harzburgite system, indicating that the multiple saturation point shifts to higher pressures and lower temperatures under hydrous conditions^41^. However, if the hydrous melt were unable to escape from the refractory segment (i.e., in a closed system), the H_2_O could be retained. Even if the hydrous melt were able to escape, it would have ascended into a magma chamber containing more abundant melt derived from the MORB source. This hydrous melt would contain only small amounts of trace elements because of its refractory source material, and would have been further diluted by the MORB melt. Therefore, it is difficult to determine its geochemical signatures in later-erupted MORB. This likely explains why sources enriched in H_2_O, such as the Horoman peridotites, have not been identified in many geochemical studies of MOR magmas.

The Horoman data suggest that ancient oceanic mantle domains, which involved highly unradiogenic Pb isotopes in sulfide formed by SSH alteration following melt extraction at an MOR, have survived in the mantle for at least 1 Gy, and that they have also retained the SSH-alteration-associated enrichments in fluid-mobile elements and H_2_O. Our results provide direct evidence for the deep subduction of hydrated oceanic mantle, which could carry fluid-mobile and volatile elements and ancient seawater (the latter at wt.% levels) into the mantle. This larger amount of H_2_O subduction, related to seafloor hydration of oceanic lithospheric mantle, indicates higher H_2_O concentrations in the mantle than indicated based on basalt chemistry^11^. If the refractory mantle segments repeatedly cycle over MORs, carrying H_2_O into the deep Earth via subduction, H_2_O storage within the mantle could increase considerably over a period of circa 1 Ga (Supplementary Discussion). Increasing the proportion of refractory but hydrous domains within the mantle could affect the rheology of the mantle, perhaps enhancing convection^11^. Moreover, the deep subduction of sulfide-bearing hydrothermally altered oceanic mantle could contribute to the production of unradiogenic Pb isotope reservoirs with lower bulk-rock U/Pb ratios^40^.

The SSH alteration could have become a common geological process, even in the Eoarchean era^42^ when modern-style plate tectonics was already in operation^43^. During the early to middle Neoproterozoic, subduction-zone geotherms appear to have become significantly lowered, enabling the oceanic lithosphere hydrated by SSH alteration to transport water into the mantle^44^. If SSH-altered oceanic mantle peridotites have been subducted and recycled in the mantle since the Neoproterozoic, then ancient, hydrous, refractory mantle domains must exist ubiquitously (estimated at 20%–30% of the upper mantle) within the present mantle^17,35^. Furthermore, their presence must have direct impact on geodynamic and seismic models of the mantle, which might require model re-evaluation. We suggest that the chemical composition of the mantle should be reconsidered more broadly, taking into account the contribution from hydrous, refractory mantle domains.

## Methods

All the analyses and experiments were performed at the Pheasant Memorial Laboratory for Geochemistry and Cosmochemistry, Institute for Planetary Materials, Okayama University at Misasa, Japan. Analytical results for 22 MSP and 10 TLP samples, and 5 gabbroic and 5 metasedimentary samples are presented in Supplementary Tables S1, S2 and S3; other data generated or analyzed during this study are included in Supplementary Table S4. The major and trace element abundances and the Nd-Hf-Pb isotope systematics of the peridotite samples had all been examined previously^17,18^, and the sample powders used in this study were the same as those analyzed in the earlier studies.

### Textural observations and *in situ* analyses

Observations using thin sections of rock samples and resin mounts of experimental products, and qualitative element abundance analysis were conducted using a JEOL JSM-7001F scanning electron microscope (SEM) equipped with energy dispersive X-ray spectrometers (EDX) and Oxford INCA X-Max and X-act. The analyses were conducted under conditions of 10 kV acceleration voltage, 3 nA beam current, and 100 s integration time. Modal abundances of minerals were obtained using X-ray maps for Na, Mg, Al, Si, S, Ca, Ti, Fe, and Cr obtained with a JEOL JXA-8800 electron probe microanalyzer (EPMA). X-ray mapping was performed with an accelerating voltage of 15 kV, beam current of 500 nA, beam diameter of 10 µm, dwell time of 20 ms, pixel spacing of 15–20 µm, and mapping area of 3–6 cm^2^. The X-ray maps were processed using Adobe Photoshop^®^ software, and the modal abundances of minerals were measured by counting the numbers of pixels of the processed binary image with a detection limit of 0.005 vol.%.

Concentrations of Li were determined by secondary ion mass spectrometry (SIMS) using a modified Cameca ims-5f ion microprobe^45^. Samples were coated with 30 nm of gold to avoid charging. The primary O^−^ beam was accelerated at −12.5 keV to impact samples with an energy of −17 keV. Secondary ions were positively accelerated at +4.5 keV. The secondary ions were counted with an electron multiplier in magnetic-peak-jumping mode. The beam intensity for Li, *I*(^7^Li^+^), relative to that of the reference element ^30^Si, was measured to determine concentrations. The integration times for ^7^Li^+^ and ^30^Si^+^ were 10 and 5 s in each cycle, respectively, and each run consisted of 6 cycles. Resulting craters were approximately 10 μm in diameter. Concentrations of Li were estimated by [Si] on predetermined sites obtained by either SEM-EDX or EPMA in advance and relative ion yield, *Y*. The value of *Y*, expressed as *Y* ≡ {*I*(^7^Li^+^)/*I*(^30^Si^+^)}/([Li]/[Si]), was obtained by analyzing a series of reference materials. The typical analytical error was <10%, based on the reproducibility of repeated analyses of reference materials. Element concentrations in the reference materials, including homogenized volcanic glass (gl-tahiti: [SiO_2_] = 59.0 wt.%, [Li] = 20.8 ppm), natural olivines (ol-sc1: [SiO_2_] = 40.6 wt.%, [Li] = 1.3 ppm; ol-fo1: [SiO_2_] = 41.5 wt.%, [Li] = 29 ppm), were determined by wet chemistry, as described later.

The isotope compositions of Li were determined by high-resolution SIMS using a modified Cameca ims-1270^46^. The sample, coated with 30 nm of gold, was sputtered with a primary O^−^ beam accelerated to −23 keV. The primary beam current was varied from 0.5 to 5.0 nA to maintain constant secondary ion intensity. Ion optics were set to achieve mass resolution power of 1200, sufficient to eliminate interference from ^6^LiH^+^. The intensity ratio of ^6^Li^+^ and ^7^Li^+^ was determined in magnetic-peak-jumping mode by ion counting with an electron multiplier. The integration times for ^6^Li^+^ and ^7^Li^+^ were 4 and 1 s in each cycle, respectively, and each run consisted of 110 cycles. Crater size was <15 μm in diameter. The ion intensity ratios obtained are expressed as deviations from the isotope ratio of the Li-isotope standard, NIST RM8545 (LSVEC); δ^7^Li^+^_SIMS_ = ([^7^Li^+^/^6^Li^+^]/[^7^Li/^6^Li]_LSVEC_ − 1) × 1000, where [^7^Li/^6^Li]_LSVEC_ = 12.1163 ± 0.0098 (2σ)^47^. Instrumental mass fractionation was corrected using the relationship δ^7^Li = δ^7^Li^+^_SIMS_ + *k*_IMF_, where *k*_IMF_ is a correction factor estimated using a reference forsterite (ol-fo1: [Li] = 29 ppm, δ^7^Li = 5.6). The typical analytical error on δ^7^Li obtained in this study was approximately 2‰ (in 2σ level), based on repeated analysis of the reference forsterites ol-fo1 and ol-fo2. The matrix effect derived from a variety of chemical compositions of olivine (e.g., Fo ≡ 100 × [MgO]^mol^/{[MgO]^mol^ + [FeO]^mol^}), suggested by ref.^48^, was evaluated by analyzing reference olivines with different compositions (ol-sc1: Fo90, [Li] = 1.3 ppm, δ^7^Li = 2.9; ol-fo1: Fo99, [Li] = 29 ppm, δ^7^Li = 5.6; ol-fo2: Fo99, [Li] = 23 ppm, δ^7^Li = 10.6; ol-fa1: Fo1, [Li] = 29 ppm, δ^7^Li = 14.8). As a result, the matrix effect was estimated as ∆δ^7^Li/∆Fo = 0.11; in cases analyzing the olivines with Fo80-Fo100 using the reference olivine, ol-sc1 (Fo90), deviations of ±1.1‰ were expected from the matrix effect. However, such deviations are smaller than the typical analytical errors on our Li isotope analysis. Thus, we utilized the reference forsterites (ol-fo1 and ol-fo2; Fo99) for the analysis of synthetic forsterite (Fo100), but the reference olivine (ol-sc; Fo90) was used for analyses of the run products of the experiment BEP1001, without matrix effect correction.

### Whole-rock analyses

Analyses were duplicated, except for Sr isotopes, and average values from these duplicate analyses are presented in this paper. Element abundances of Si, Ti, Al, Fe, Mn, Mg, Ca, P, Cr, and Ni of starting materials for synthetic experiments were determined by X-ray fluorescence spectrometry using a Philips PW2400. Powdered samples (0.5 g) were mixed with Li_2_B_4_O_7_ (5 g). The instrument was calibrated using GSJ reference materials^49,50^. Loss-on-ignition was determined gravimetrically. Powdered samples (0.2–0.4 g) were heated at 1000 °C for 4 h, and then weighed using a balance. Boron abundances of the samples were determined by ICP-QMS applying the isotope dilution method^51^. Analytical uncertainty (RSD) during the analyses was 2.5% (n = 9). Sulfur concentrations were determined by oxidation of S into sulfate with *in situ* generation of Br_2_ using the isotope dilution high-resolution ICP-MS method^52^. Analytical uncertainty (RSD) during these analyses was 9% (n = 3). Concentrations of F and Cl were measured using pyrohydrolysis and ion chromatography following the procedure described in ref.^53^. A GSJ reference material, JB-3, was measured during the analyses. The analytical uncertainties (RSD) for F and Cl were 3.6% and 2.4% (n = 5), respectively.

Elemental and isotope abundances of Li were determined by almost the same methods as described in ref.^54^, except that Li isotope measurements were carried out using a MC-ICP-MS, Thermo-Finnigan Neptune^55^. Lithium isotope compositions are represented as deviations from that of the NIST LSVEC; δ^7^Li = ([^7^Li/^6^Li]/[^7^Li/^6^Li]_LSVEC_ − 1) × 1000, where [^7^Li/^6^Li]_LSVEC_ = 12.1163^47^. During this study, δ^7^Li values for a GSJ standard sample, JB-2 (δ^7^Li = 4.8‰ ± 0.5‰^56^), were determined twice, which resulted in consistent values of 4.84‰ ± 0.34‰ (n = 6, 2σ) and 5.03‰ ± 0.52‰ (n = 15, 2σ). Chemical separations of Rb and Sr were achieved in the same way described in refs^57,58^. Isotope measurements were made using a Thermo-Finnigan TRITON thermal ionization mass spectrometer (TIMS) for ^87^Sr/^86^Sr ratios, whereas for Rb concentrations, a Finnigan MAT 262 TIMS was employed. Static multicollection mass spectrometry was undertaken for the simultaneous determination of concentrations and isotope compositions of Sr by isotope dilution mass spectrometry^58^. During this study, total blanks (n = 4) for Rb and Sr were 1 and 27 pg, respectively. Measured abundances of Rb and Sr, and the ^87^Sr/^86^Sr ratios in GSJ JB-2 were 6.30 ppm, 179 ppm, and 0.703690 ± 0.000005 (n = 4, 2σ_m_), and the ^87^Sr/^86^Sr ratios in NIST SRM987 were 0.710268 ± 0.000016 (n = 10, 2σ_m_). The isotope age was calculated using Isoplot (Ex version 4.15)^59^ and errors on both the calculated age and the initial isotope ratio are quoted at the 95% confidence level. Purifications of Nd and Sm were carried out using the methods described by ref.^57^, and their abundances were determined by TIMS (Finnigan MAT 262 and Thermo-Finnigan TRITON) for Nd and by the MC-ICP-MS for Sm. The Ti-addition method^60^ was employed for purification of Hf and Lu, and the isotope ratios of Hf and Lu were determined using the MC-ICP-MS^60,61^. Normalizing values for isotope fractionation corrections for ^143^Nd/^144^Nd and ^176^Hf/^177^Hf were 0.7219 and 0.7325, respectively.

H_2_O contents and δD values were determined using 3–36 mg samples and a thermal conversion elemental analyzer/gas-source mass spectrometer, employing a method modified after ref.^62^. The measured H_2_O content of Kamagaya reference sericite was 6.2 ± 0.1 wt% (0.621–1.29 mg, n = 12, 1σ). The measured δD of the standard materials was −78.4‰ ± 2.8‰ for an in-house-reference MSP1 (0.057–0.184 mg, n = 15, 1σ) and −114.6‰ ± 0.8‰ for NBS22 (0.007–0.09 mg, n = 11, 1σ). Oxygen isotope ratios of 1–2 mg peridotite whole-rock powders were determined by CO_2_ laser fluorination, in the presence of BrF_5_, and by gas-source mass spectrometry^63^. Isotope ratios were measured using a Finnigan MAT253 mass spectrometer^64^. The ^18^O/^16^O of the working standard gases was calibrated by VSMOW-extracted O in each fluorination-extraction line. During this study, the δ^18^O_VSMOW_ value obtained for the in-house garnet standard MSG-1 was 5.96‰ ± 0.06‰ (n = 18, 1σ), and that for San Carlos olivine was 5.25‰ ± 0.03‰ (n = 5, 1σ).

## Electronic supplementary material

Supplementary Information
