# Supplementary material for: Circa 1 Ga sub-seafloor hydrothermal alteration imprinted on the Horoman peridotite massif
Source: Sci Rep. 2018 Jun 29;8:9887. doi: 10.1038/s41598-018-28219-x (PMC6026181; doi:10.1038/s41598-018-28219-x)
Supplement: Supplementary file 1 — Supplementary Information [file 41598_2018_28219_MOESM1_ESM.pdf]

SUPPLEMENTARY INFORMATION

Circa 1 Ga sub-seafloor hydrothermal alteration imprinted on Horoman peridotite massif

Lalindra V. Ranaweera[1,2], Tsutomu Ota[1], Takuya Moriguti[1], Ryoji Tanaka[1], and Eizo Nakamura[1]

[1] Pheasant Memorial Laboratory for Geochemistry and Cosmochemistry, Institute for Planetary Materials, Okayama University at Misasa, Tottori 682-0193, Japan.
[2] Department of Natural Resources, Faculty of Applied Sciences, Sabaragamuwa University of Sri Lanka, Belihuloya, Sri Lanka.

* Correspondence and requests for materials should be addressed to E.N. (e-mail; eizonak@misasa.okayama-u.ac.jp)

Contents

Supplementary Experiment
– Li-isotope fractionation between olivine and fluid
– Synthetic experiment
– Lithium isotope variation of the Horoman peridotites
Supplementary Discussion
– Water content in the mantle
Supplementary References
Supplementary Figures (S1–S6)
Supplementary Tables (S1–S4)

Supplementary Experiment

Li-isotope fractionation between olivine and fluid

Parts of the mantle lithologies are hydrothermally altered at sub-seafloor, varied in elemental and isotope abundances of Li[28], and then subduct into deep mantle. Then, the issues are how Li-isotope abundances in mantle lithologies are modified through fluid-related processes at sub-seafloor and at depths in subduction zones, and by what Li-isotope abundances `recycled' mantle lithologies are characterized. Observation of modern serpentinized peridotites from the MOR[28] and the forearc[29] settings suggest that the serpentinisation would modify Li-isotope abundances of the mantle lithologies to be depleted in ^{7}Li, compared with the primitive mantle; it has been verified by experiments on the serpentine-fluid Li-isotopic fractionation[32]. However, another experiment to examine the Li-isotope fractionation in an olivine-clinopyroxene-fluid system[33] have included inconsistent results, probably because of low-temperature experiments in short duration (at 800 °C for 68-72 hours). One of the experimental products at 800 °C (2m-2 olivine in ref. 33) is described as olivine, but its major element composition is rather similar to that of serpentine (Table 4 in ref. 33), although serpentine minerals should be unstable under the experimental conditions of 800 °C[36]. This result implies that not only this experiment but also other experiments at 800 °C could have been in disequilibrium. They presented another data on the olivine at 1000 °C, being consistent with that in the clinopyroxene-fluid system[30], but temperature dependence of the Li-isotope fractionation between olivine and fluid has been unclear, yet. Thus, we conducted supplementary experiments in a Mg_2SiO_4 (forsterite) + H_2O system, under upper mantle conditions.

Synthetic experiment

The experiment was conducted using a piston cylinder apparatus. Starting materials were prepared by mixing high-purity reagents of Li_2SiO_3, SiO_2, MgO, and $Mg(OH)_2$. These were ground with acetone in an agate mortar, dried in an oven at 110 °C, and loaded into a Pt capsule, which was then sealed by welding. A half-inch-diameter assembly with a straight graphite heater, and the hot piston-out method, were applied. Experimental conditions of 2 GPa and 900 °C were maintained for 24–110 h. By shutting off the heater power supply, quenching to room temperature was achieved within 1 min, and the pressure was then released to atmospheric pressure. The recovered Pt capsules were first cleaned by removing

pieces of the assembly, weighed, then perforated using a steel needle to check for the release of liquid water, indicating the amount of fluid present during the experiment. Then, the capsule was dried at 110 °C to evaporate any liquid water, and weighed to calculate the amount of H_2O retained within the capsule during the experiment. Finally, if the calculated H_2O content in the recovered capsule was consistent (±20%) with that in the starting material loaded into the capsule, we regarded the experiment successful. Then, the capsule was mounted in epoxy resin, polished, carbon-coated, and observed by using SEM to confirm run products and to examine textures. After the SEM observation, the resin mount was polished to remove carbon, and gold-coated for the SIMS analysis to determine elemental and isotope abundances of Li in the run product. Based on elemental and isotope abundances of Li in the starting material and in the run product, the isotope fractionation of Li between the run product (olivine) and fluid, $\Delta^7Li_{olivine\text{-}fluid} = \delta^7Li_{olivine} - \delta^7Li_{fluid}$, was estimated based on mass balance.

All experiments yielded only forsteritic olivine as solid phase. Most of the olivine is euhedral, and ranges in size from 20 to 100 μm, and fine-grained crystals were predominant in run products synthesized in short durations. Elemental abundances of Li in the olivines are homogeneous within a grain, but vary significantly among grains, particularly in the run products of short-duration experiments (BE1001 and BEP1001). On the other hand, an experiment BEP1003 with the long run duration yielded coarse-grained olivines, grown to 150 to 200 μm in long-axis length. Nevertheless, the olivines synthesized are homogeneous in elemental and isotope abundances of Li both within a grain and among grains, compared with those from other experiments in short run durations. The long-duration experiments (for 110 hours), BE1003 and BEP1003, yielded olivines with less scattered elemental and isotope abundances of Li than those synthesized in shorter duration experiments, which exhibited significant deviations in elemental or isotope abundances larger than analytical uncertainties (10 % in RSD or 2 ‰ in 1σ). And the olivines from experiments BE1003 and BEP1003 are similar in elemental and isotope abundances of Li to each other though the starting materials of these experiments are different in H_2O and Li-isotope abundances. Thus, we regarded these two experimental run products as the most reliable one to estimate the Li-isotope fractionation, being $\Delta^7Li_{olivine\text{-}fluid} = -2 \pm 2$ ‰ (**Supplementary Fig. S5** and **Table S4**). This result is consistent with the temperature-dependent Li-isotope fractionation for silicate minerals and fluids[30-33], and implying that the `recycled' mantle lithologies could become more depleted in ^7Li than the primitive mantle.

Lithium isotope variation of the Horoman peridotites

Significant bulk-rock δ^7Li variation can be produced by diffusion-induced isotope fractionation during infiltration of rocks by Li-rich melts or fluids[76,77]. Recently, it was argued that the Li isotope compositions of the Horoman peridotites were locally modified by kinetic isotope fractionation during diffusive ingress of Li in externally sourced melts[78], as the peridotites, which are more depleted in incompatible elements than the PL-MSPs, show enrichment in ^{6}Li coincident with enrichments in the highly incompatible elements such as La and Sr. However, the PL-MSPs that we have examined show negligible element enrichment by melt-related metasomatism[17] and exhibit undetectable correlation of δ^7Li with the enrichments of La and Sr, and other incompatible elements (**Supplementary Fig. S6**).

Previous studies on the Li-isotope diffusion indicate that clinopyroxene inherits very low δ^7Li values through faster ^{6}Li diffusion, whereas olivine is more resistant to diffusive modification[76,77]. If the anomalously low bulk-rock Li-isotope variation results from kinetic fractionation by the diffusive Li-ingress, clinopyroxene is expected to be lower in δ^7Li value than olivine. However, among the Li-isotope analyses presented by ref. 78, the δ^7Li values of minerals, which are separated from the peridotites showing the anomalously low bulk-rock δ^7Li values, are the lowest in olivine. On the other hand, experimental studies on Li-isotope fractionation in isotopically equilibrated systems have demonstrated that ^{6}Li prefers the coordination state higher than that of the fluid phase, that is, [6]-fold coordinated sites in most ferromagnesian minerals including pyroxenes, olivine, and serpentine[30-33]. When serpentine breaks down to release aqueous fluid during recycling of the serpentinized abyssal peridotites in the mantle[36], the resultant olivine would be enriched in ^{6}Li, as suggested by the experiments in an olivine-clinopyroxene-fluid system[33] and in an olivine-fluid system (this study).

Consequently, it is unlikely that diffusion-induced kinetic fractionation is a predominant mechanism in the generation of the Li isotope variation of the PL-MSPs. The low δ^7Li values (< 2) of the PL-MSPs are considered to be the evidence that the Horoman peridotites preserve chemical signatures of the ancient SSH alteration at a MOR (enrichments of fluid-mobile and volatile elements), the recent fluid-interactions at subduction-zone settings (enrichments of the fluid-mobile elements including Pb enriched in its radiogenic isotopes, and Rb leading enriched Sr-isotope compositions), and the dehydration reactions during subduction into the mantle (fractionated H-Li isotope compositions).

Supplementary Discussion

Water content in the mantle

A retention of 0.20 to 0.58 cg/g H_2O in peridotites with merely no alteration phases (i.e. DP-MSP) is significant because H_2O solubility of mineral phases in the upper mantle like orthopyroxene is considered to be in trace amount[79]. According to experimental and thermodynamic data of MgO-SiO_2-H_2O system, serpentine (antigorite) in a hydrous peridotite with bulk 12.2 cg/g of water is stable up to approximately 5 GPa, and become *phase A* – a dense hydrous magnesium silicate and enstatite, beyond that with approximately 4 cg/g of bulk water which is stable up to mantle transition depths[36]. If serpentine was the dominant hydrous phase formed in the Horoman peridotites as the result of SSH alteration at a MOR and bending-related faulting at trench–outer rise, like hydrothermally-altered peridotites at modern sub-seafloor[5], it would have been transferred into (forsterite + enstatite + fluid) or (*phase A* + enstatite + fluid) in a cold subduction-zone geotherm, and (*phase A* + enstatite) would decompose into (forsterite + fluid) during the mantle convection at a nominal mantle geotherm[36]. From this perspective, the altered peridotite is regarded as one of the primary carriers of H_2O in subducting lithosphere into the mantle[34,36,80].

Given the total length of mid-ocean ridges (approximately 6.7×10^4 km; ref. 4) with assuming minimum 20% of ridge hydrothermal alteration[4] up to 5 km depth (estimated present thickness of the Horoman mantle is 3.7 km; ref. 12) and assuming 0.2 to 0.6 cg/g water, based on our data, in a subducting oceanic mantle with an average plate velocity of 46.6 mm/y, it delivers water approximately 10^{10} kg/y into the mantle. Altered oceanic crust also delivers water approximately 10^{15}-10^{16} kg/y into the subduction zones, and there would be about factor of 10 difference net flux of water into the mantle[81]. Basalts extracted at ridges and oceanic islands consistently suggest low water contents (50-1000 μg/g) in their source regions of the mantle[11], and the global flux of water out gassed from the Earth's interior is in the order of 10^{11} kg/y. Thus, the amount of outgas flux of water from the mantle seems to be less than the influx of water. Our value gives a minimum estimate, and therefore, the water content in the mantle appears to be higher than the present estimate (approximately 10^{21} kg; ref.11).

Supplementary References

76. Parkinson, I., Hammond, S., James, R., & Rogers, N. High-temperature lithium isotope fractionation: Insights from lithium isotope diffusion in magmatic systems. *Earth Planet. Sci. Lett.*, **257**, 609-621 (2007).

77. Rudnick, R. L. & Ionov, D. A. Lithium elemental and isotopic disequilibrium in minerals from peridotite xenoliths from far-east Russia: Product of recent melt/fluid-rock reaction. *Earth Planet. Sci. Lett.* **256,** 278-293 (2007).

78. Lai, Y.-J., Pogge von Strandmann, P. A. E., Dohmen, R., Takazawa, E., & Elliott, T. The influence of melt infiltration on the Li and Mg isotopic composition of the Horoman Peridotite Massif. *Geochim. Cosmochim. Acta* **164**, 318-332 (2015).

79. Mierdel, K., Keppler, H., Smith, J.R. & Langenhorst, F. Water solubility in aluminous orthopyroxene and the origin of Earth's asthenosphere. *Science* **315,** 364-368 (2007).

80. Hatakeyama, K., Katayama, I., Hirauchi, K.-I., & Michibayashi, K. Mantle hydration along outer-rise faults inferred from serpentinite permeability. *Sci. Reports* **7(1)**, 13870, (2017).

81. Bebout, G. E. Volatile transfer and recycling at convergent margins: mass-balance and insights from high-P/T metamorphic rocks. *AGU Geophys. Monogr.* **96,** 179-193 (1996).

82. Schmidt, K., Koschinsky, A., Garbe-Schönberg, D., M. de Carvalho, L. & Seifert, R. Geochemistry of hydrothermal fluids from the ultramafic-hosted Logatchev hydrothermal field, 15° N on the Mid-Atlantic Ridge: Temporal and spatial investigation. *Chem. Geol.* **242,** 1-26 (2007).

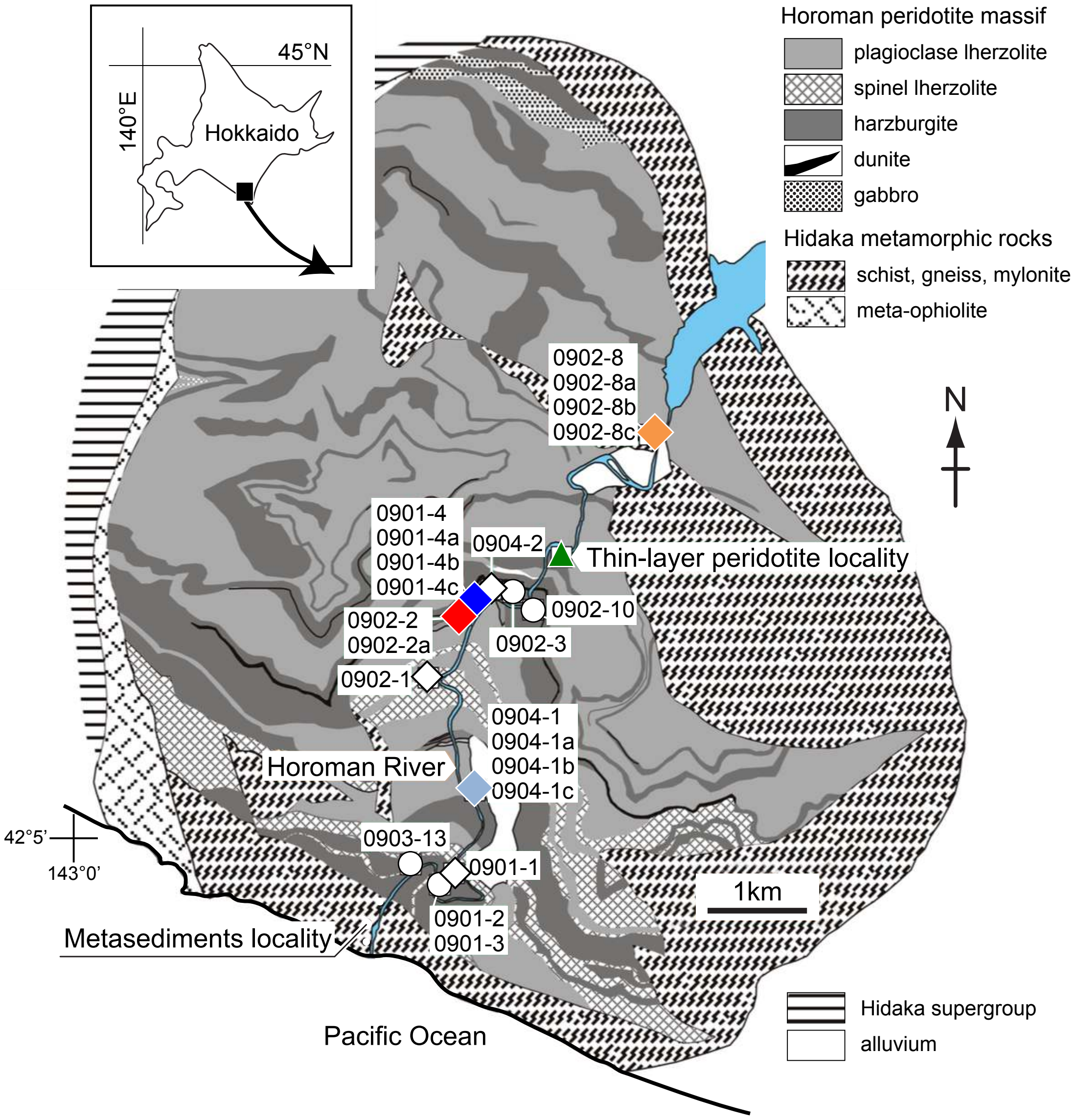

Figure S1. Lithologic map of the Horoman peridotite massif. The map is modified after ref. 75. Localities of samples examined in this study are also shown, with symbols as the same as in Fig. 2. A series of samples, such as 0902-8, 0902-8a, 0902-8b, and 0902-8c, are collected within a few meters of an outcrop.

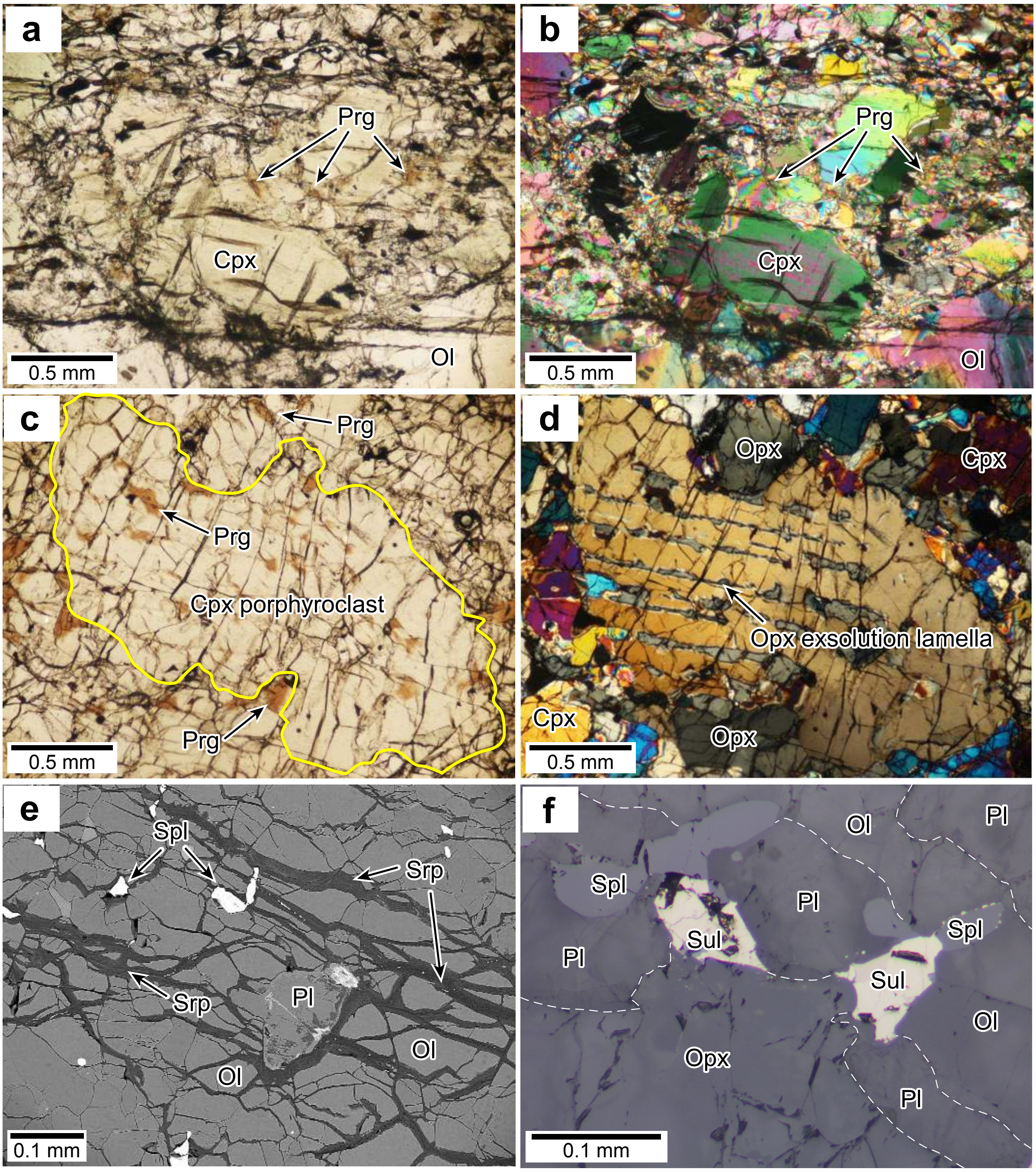

Figure S2. Modes of occurrence of hydrous minerals and sulfide in the Horoman peridotites. a, Photomicrograph of pargasite (Prg), associated with clinopyroxene (Cpx) porphyroclasts, and occurring in fine-grained matrix (sample, 0904-1). **b**, Cross-polarized light view of **a**, showing porphyroclastic texture where Prg has subjected to grain-size reduction. **c**, Photomicrograph of patchy Prg in Cpx porphyroclast, and its grain boundary (traced by yellow line), suggesting its metasomatic origin either by melt or fluid (sample, HR-70). **d**, Cross-polarized light view of **c**, showing orthopyroxene (Opx) lamellae with patchy Prg in the porphyroclast. **e**, Backscattered electron image of mesh-textured serpentine (Srp) in olivine (Ol) in the sample (0902-8c), with the highest modal abundance of Srp among the studied ones. **f**, Photomicrograph under a reflected-light, of discrete anhedral sulfide (Sul), filling grain boundaries among surrounding phases (sample, 0902-2a).

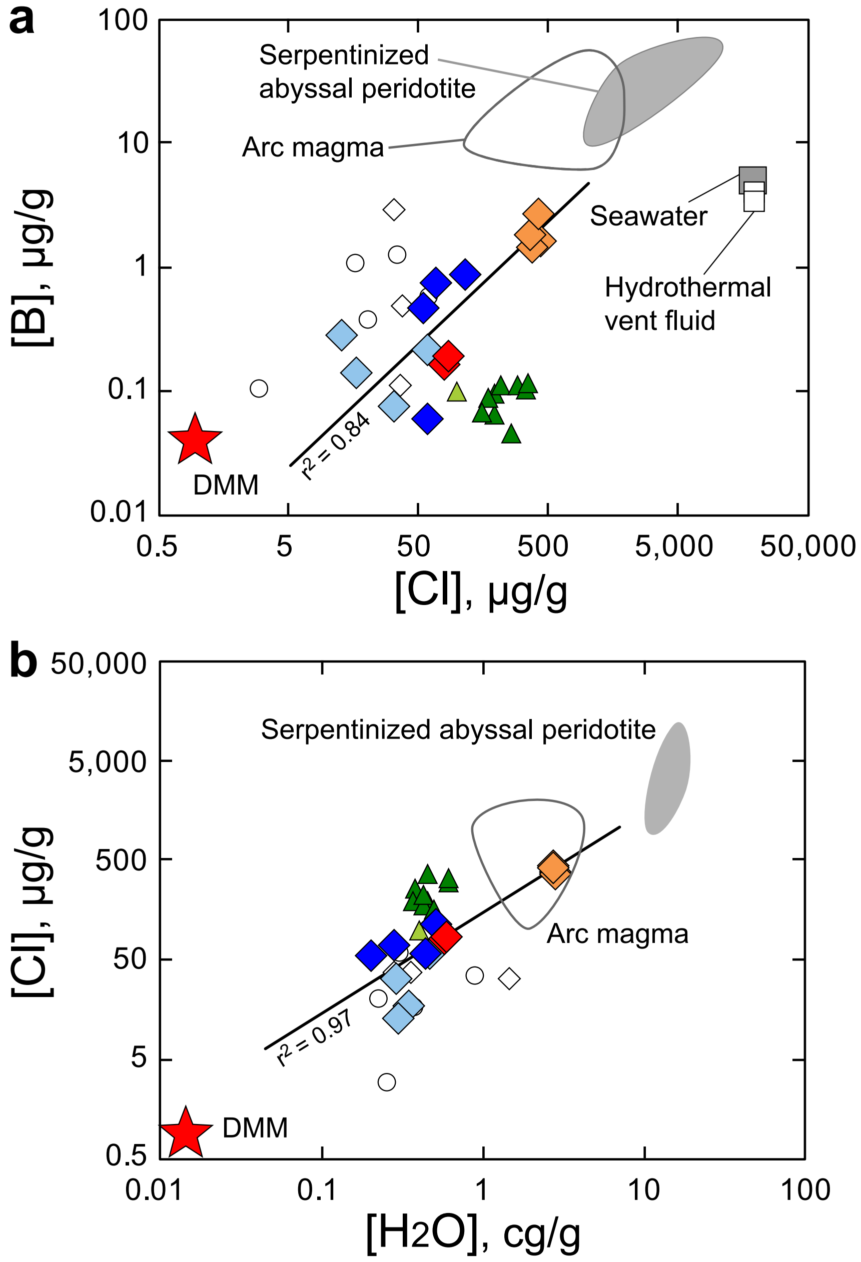

Figure S3. Elemental abundances of B, Cl and H_2O of the Horoman peridotites. Regression lines for data of the massive plagioclase-lherzolites are shown. Symbols are the same as in Figs. 2 and S1. Analytical uncertainties (2σ) are 5.0, 4.8, and 3.2 %, for B, Cl, and H_2O, respectively, and their corresponding error bars are smaller in size than individual symbols. Data sources of DMM, arc magma, serpentinized abyssal peridotite, and seawater and hydrothermal vent fluids are refs. 3, 67, 73, and 82, respectively.

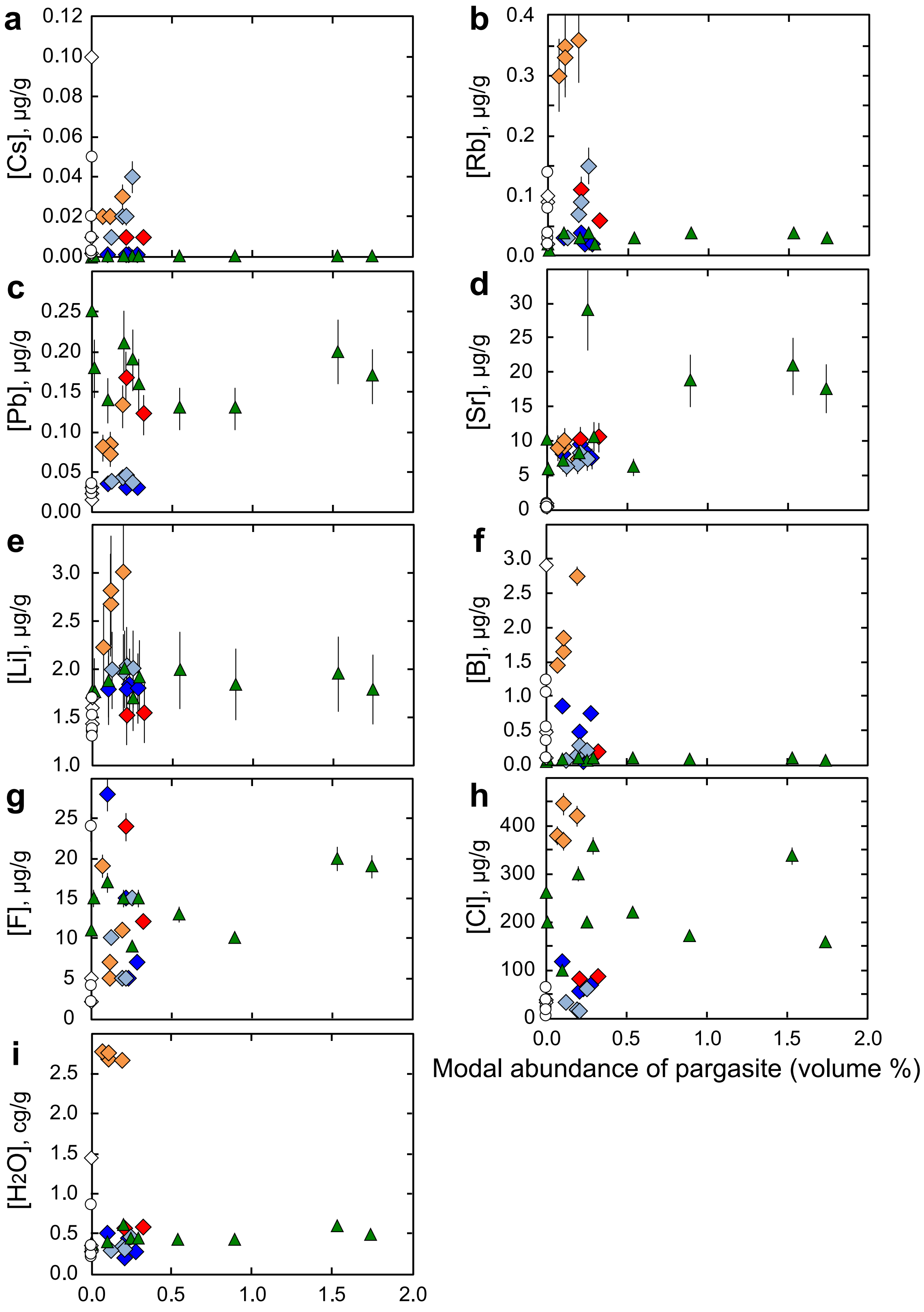

Figure S4. Modal abundance of pargasite versus elemental abundances of the Horoman peridotites. Elements concerned are **a**, Cs, **b**, Rb, **c**, Pb, **d**, Sr, **e**, Li, **f**, B, **g**, F, **h**, Cl, and **i**, H_2O. Elemental abundances of Cs, Rb, Pb, Sr, and Li are taken from previous studies[17,18]. Symbols are the same as in Figs. 2 and S1. Error bars denote analytical uncertainties (2σ) for elemental abundance, and are smaller than symbols in some cases.

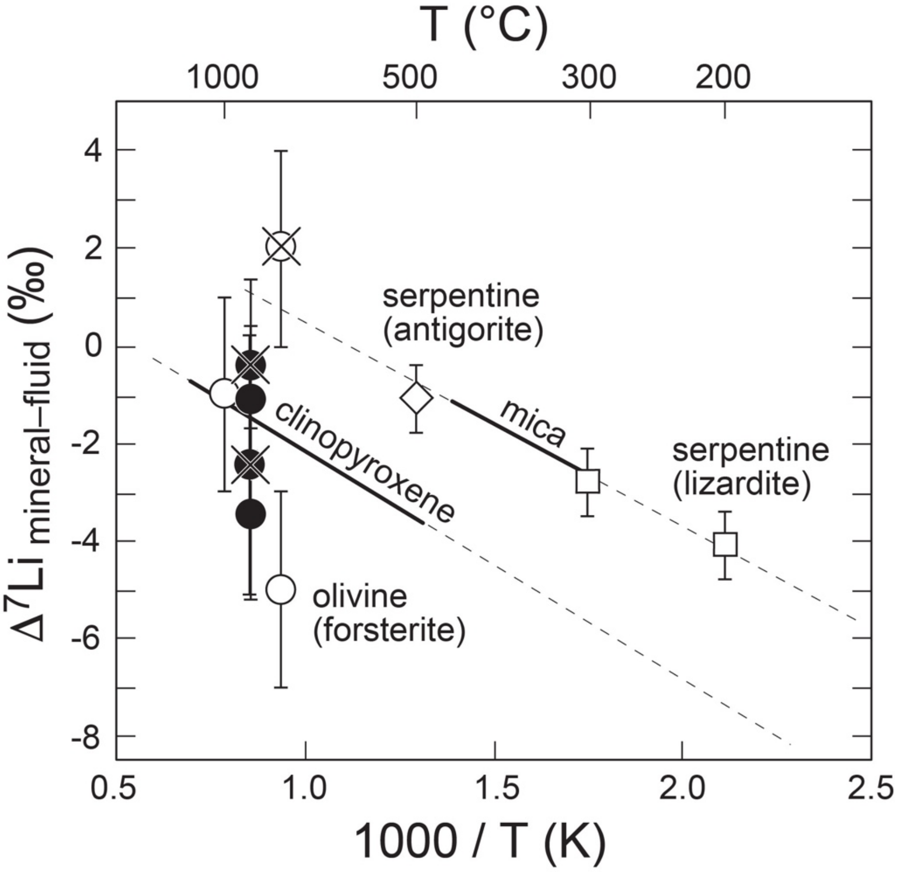

Figure S5. Experimentally determined Li-isotope fractionation between silicate minerals and fluids as a function of temperature. Error bars correspond to 1σ uncertainty on Li isotope analyses on the minerals in experimental run products. Data are shown for clinopyroxene-fluid[30,33] and for Li-bearing mica-fluid[31] with solid lines, for Li-bearing serpentine-fluid with open rectangular[32], for Li-bearing olivine-fluid with open circle[33] and with solid circle (this study). One of the data for Li-bearing olivine-fluid at 800 °C (open circle with cross) is derived from a phase different from olivine according to descriptions about the run product (2m-2 olivine) in ref. 33, and two of the data in this study (solid circle with cross) are derived from the experiments (BEP1001 and BEP1002) that could have not achieved chemical equilibrium. Therefore, these data, shown with crossed symbols, were excluded in discussion.

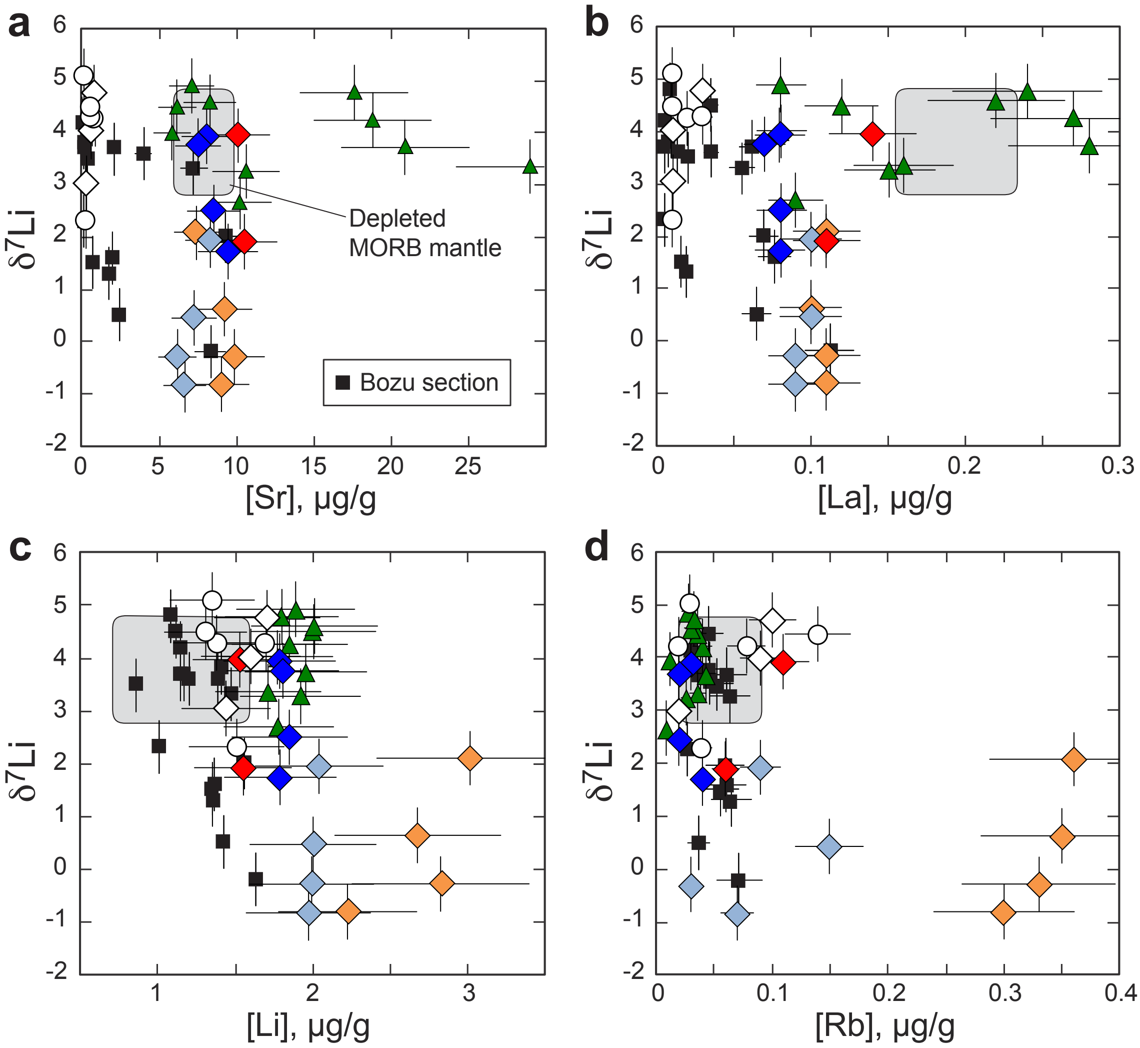

Figure S6. Elemental abundances of incompatible and fluid mobile elements versus isotope abundance of Li of the Horoman peridotites. The incompatible elements concerned here are **a**, Sr, and **b**, La, and the fluid mobile elements are **c**, Li, and **d**, Rb. Data plotted include those taken from previous studies (Horoman river section[17,18] and Bozu section[15,78]), and their symbols are the same as in Figs. 2 and S1, except solid squares indicating data from the Bozu section. Error bars show 2σ-uncertainties on elemental (< 20 %[17,18], 1.3 %[78], or 12-27 %[15]) and isotope (0.52 ‰ or 0.5 ‰[78]) abundance analyses. Data of depleted MORB mantle are after refs. 3, 65, and 70.

Table S1. Modal abundances of minerals in the Horoman peridotites.

Rock name	Sample name	Ol	Opx	Cpx	Pl	Spl	Prg	Srp	Sulfide
Massive peridotite									
Plagioclase-lherzolite	0901-4 series								
	0901-4	59.4	20.0	11.1	8.38	0.80	0.23	< 0.01	0.04
	0901-4a	50.9	33.8	7.89	6.85	0.39	0.10	< 0.01	0.03
	0901-4b	64.3	17.6	8.82	8.72	0.30	0.21	< 0.01	0.03
	0901-4c	64.0	22.6	6.31	6.30	0.50	0.28	< 0.01	0.04
	0902-2 series								
	0902-2	72.9	17.3	5.18	3.88	0.48	0.20	< 0.01	0.08
	0902-2a	55.9	20.8	10.4	11.9	0.69	0.32	< 0.01	0.05
	0902-8 series								
	0902-8	57.4	6.30	10.2	6.27	0.56	0.11	19.1	0.04
	0902-8a	59.0	8.80	9.16	3.31	0.43	0.07	19.2	0.06
	0902-8b	35.2	31.4	7.22	8.08	0.85	0.11	17.1	0.07
	0902-8c	53.4	8.50	7.37	9.33	0.77	0.19	20.4	0.07
	0904-1 series								
	0904-1	70.8	19.2	4.01	4.53	1.32	0.19	< 0.01	0.02
	0904-1a	68.4	17.4	9.66	3.26	0.97	0.21	< 0.01	0.06
	0904-1b	66.7	17.0	6.31	9.12	0.74	0.12	< 0.01	0.03
	0904-1c	76.0	10.2	9.33	3.02	1.24	0.25	< 0.01	0.01
Spinel-lherzolite	0901-1	60.9	23.8	10.3	< 0.01	2.27	< 0.01	2.82	< 0.01
	0902-1	52.0	32.2	11.5	< 0.01	2.11	< 0.01	2.09	0.02
	0904-2	63.7	18.2	8.27	< 0.01	0.66	< 0.01	9.12	0.03
Spinel-harzburgite	0901-2	78.8	14.8	3.14	< 0.01	0.44	< 0.01	2.86	< 0.01
	0901-3	85.1	8.80	1.30	< 0.01	2.43	< 0.01	2.38	< 0.01
	0902-3	72.3	20.0	4.53	< 0.01	2.28	< 0.01	0.95	< 0.01
	0902-10	78.9	14.2	3.54	< 0.01	0.39	< 0.01	2.97	< 0.01
	0903-13	77.0	14.8	3.52	< 0.01	0.36	< 0.01	4.26	< 0.01
Thin-layer peridotite									
Plagioclase -lherzolite	HR+55	57.5	26.2	8.53	5.99	0.76	0.25	0.77	0.05
	HR+20	60.3	24.4	7.90	5.51	0.65	< 0.01	1.21	0.01
	HR+12	67.1	20.2	5.82	5.50	0.67	0.01	0.74	0.01
	HR-0	53.4	11.5	20.2	12.5	0.51	0.89	1.02	0.02
	HR-37	57.9	26.9	6.14	6.33	0.91	0.54	1.27	0.01
	HR-50	71.0	12.2	10.2	4.24	0.70	0.20	1.50	< 0.01
	HR-60	44.9	22.2	15.1	13.7	0.25	1.53	2.29	0.01
	HR-70	63.0	11.2	13.5	9.49	0.22	1.74	1.01	0.02
	HR-90	61.3	17.9	5.53	12.5	0.81	0.29	1.61	0.01
Plagioclase-harzburgite	HR+5	72.1	20.9	3.38	1.50	0.41	0.10	1.62	< 0.01

Modal abundances are shown in volume %. Mineral abbreviations are: Ol, olivine; Opx, orthopyroxene; Cpx, clinopyroxene; Pl, plagioclase; Spl, spinel; Prg, pargasite; Srp, serpentine.

Table S2. Elemental and isotope abundances of the Horoman peridotite.

Rock/Sample name	F	Cl	H_2O	B	S	Rb	Sr	La	Sm	Li	Pb*	δD	δ^7Li	$\delta^{18}O$	$^{87}Sr/^{86}Sr$	$^{206}Pb/^{204}Pb$	$^{207}Pb/^{204}Pb$	$^{208}Pb/^{204}Pb$	$^{143}Nd/^{144}Nd$	$^{176}Hf/^{177}Hf$
Massive peridotite																				
Plagioclase-lherzolite																				
0901-4	5	59	0.439	0.060	146	0.016	8.46	0.08	0.22	1.85	1.0	-74	2.50	5.52	0.702023	17.520	15.410	37.323	0.513674	0.283590
0901-4a	28	116	0.504	0.870	137	0.018	7.92	0.08	0.22	1.79	1.1	-72	3.94	5.36	0.702031	17.052	15.320	36.784	0.513676	0.283624
0901-4b	15	55	0.199	0.478	128	0.018	9.36	0.08	0.26	1.79	0.8	-70	1.73	5.34	0.702019	17.043	15.320	36.769	0.513684	0.283568
0901-4c	7	69	0.276	0.749	138	0.021	7.60	0.07	0.21	1.81	1.0	-75	3.76	5.26	0.702034	17.287	15.363	37.054	0.513667	0.283585
0902-2	24	81	0.572	0.168	273	0.075	10.3	0.14	0.24	1.53	3.7	-63	3.96	5.56	0.702074	16.607	15.232	36.163	0.513547	0.283535
0902-2a	12	87	0.581	0.192	156	0.070	10.8	0.11	0.25	1.55	2.7	-64	1.92	5.43	0.702057	16.666	15.237	36.233	0.513528	0.283537
0902-8	7	445	2.68	1.65	189	0.313	8.90	0.10	0.23	2.68	1.9	-85	0.63	5.30	0.702512	17.659	15.441	37.468	0.513256	0.283334
0902-8a	19	380	2.77	1.45	210	0.308	9.13	0.11	0.23	2.23	1.7	-88	-0.81	5.20	0.702435	17.629	15.431	37.432	0.513268	0.283377
0902-8b	5	368	2.75	1.85	216	0.306	10.3	0.11	0.23	2.83	1.5	-85	-0.28	5.34	0.702435	17.470	15.399	37.238	0.513285	0.283386
0902-8c	11	419	2.67	2.75	202	0.331	7.53	0.11	0.24	3.02	2.7	-83	2.10	5.34	0.702508	17.811	15.461	37.669	0.513263	0.283337
0904-1	5	17	0.340	0.144	102	0.053	6.66	0.09	0.14	1.98	1.4	-72	-0.83	5.24	0.702285	17.153	15.311	36.817	0.513096	0.283761
0904-1a	5	13	0.300	0.290	197	0.086	8.19	0.10	0.18	2.04	1.1	-60	1.95	5.27	0.702301	17.171	15.315	36.838	0.513089	0.283868
0904-1b	10	33	0.290	0.077	162	0.035	6.26	0.09	0.14	2.00	1.2	-74	-0.29	5.41	0.702296	17.095	15.309	36.761	0.513089	0.283850
0904-1c	15	59	0.460	0.216	45	0.174	7.20	0.10	0.16	2.01	0.9	-72	0.46	5.33	0.702384	17.143	15.307	36.808	0.513098	0.283811
Spinel-lherzolite																				
0901-1	2	38	0.350	0.482	27	0.071	0.531	0.01	0.02	1.60	8.7	-67	4.03	5.27	0.703470	18.330	15.549	38.318	0.513530	0.287858
0902-1	5	37	0.280	0.110	46	0.021	0.290	0.01	0.01	1.44	10	-80	3.04	5.32	0.703188	n.a.	n.a.	n.a.	0.512955	0.287037
0904-2	2	33	1.45	2.91	36	0.101	0.777	0.03	0.02	1.71	2.5	-63	4.78	5.33	0.703922	17.769	15.489	37.737	0.512847	0.288255
Spinel-harzburgite																				
0901-2	24	62	0.300	0.563	30	0.079	0.860	0.02	0.02	1.70	3.9	-62	4.26	5.30	0.703501	18.225	15.492	38.267	0.512469	0.284031
0901-3	n.d.	21	0.220	0.368	13	0.030	0.162	0.01	0.003	1.36	25	-68	5.09	5.30	0.703388	n.a.	n.a.	n.a.	0.512901	0.286872
0902-3	n.d.	3	0.249	0.104	0.7	0.019	0.620	0.03	0.005	1.39	7.8	-97	4.28	5.22	0.703720	18.687	15.612	38.755	0.512854	0.285312
0902-10	2	36	0.860	1.25	43	0.140	0.632	0.01	0.01	1.31	11	-45	4.48	5.33	0.703529	18.229	15.525	38.166	0.513114	0.290014
0903-13	4	17	0.360	1.07	5.4	0.041	0.198	0.01	0.001	1.52	17	-71	2.31	5.24	0.704733	18.521	15.605	38.543	0.512710	n.a.
Thin-layer peridotite																				
Plagioclase-lherzolite																				
HR+55	9	198	0.450	0.066	129	0.036	28.2	0.16	0.19	1.71	3.9	-79	3.36	5.42	0.702178	16.805	15.282	36.485	0.513238	0.283425
HR+20	11	260	0.380	0.046	111	0.009	9.4	0.09	0.16	1.78	7.8	-77	2.68	5.74	0.702065	16.548	15.165	36.067	0.513684	0.283997
HR+12	15	198	0.370	0.097	145	0.012	5.5	0.08	0.19	1.77	5.4	-76	4.01	5.30	0.702205	16.903	15.267	36.537	0.513395	0.283456
HR±0	10	172	0.430	0.089	79	0.040	18.4	0.27	0.57	1.85	1.1	-75	4.25	5.01	0.702360	17.596	15.370	37.331	0.513023	0.283153
HR-37	13	220	0.430	0.113	87	0.036	5.8	0.12	0.23	2.00	2.5	-82	4.50	5.20	0.702476	18.058	15.363	37.578	0.513049	0.283178
HR-50	15	299	0.610	0.114	64	0.030	7.8	0.22	0.43	2.01	2.3	-77	4.59	5.23	0.702443	18.065	15.367	37.674	0.513028	0.283110
HR-60	20	337	0.600	0.102	119	0.043	19.8	0.28	0.47	1.96	1.9	-87	3.73	5.10	0.702528	17.787	15.326	37.450	0.513076	0.283111
HR-70	19	157	0.490	0.068	179	0.033	17.3	0.24	0.50	1.80	1.8	-74	4.78	5.28	0.702477	17.762	15.362	37.454	0.513097	0.283100
HR-90	15	358	0.450	0.115	118	0.026	10.4	0.15	0.26	1.92	2.7	-75	3.27	5.27	0.702763	17.169	15.251	37.112	0.512940	0.283069
Plagioclase-harzburgite																				
HR+5	17	100	0.403	0.099	48	0.028	7.0	0.08	0.09	1.89	6.2	-85	4.91	5.40	0.702306	17.184	15.272	36.819	0.513125	0.283266

Units of elemental abundances are in μg/g, but in cg/g for H_2O.

Elemental abundance of rubidium, strontium, lanthanum, samarium and lithium, and isotope abundances of strontium, lead, neodymium, and hafnium are from ref. 17 and 18, but the abundances of which values are underlined were newly measured in this study.

Pb* = 2·Pb/(Ce + Pr), where the elemental abundances[17] are normalized to those of primitive mantle[22].

n.d.: not detected.

n.a.: not analysed.

Table S3. Oxygen and lithium isotope abundances of the Horoman gabbros and Hidaka metasediments.

Rock name	Sample name	$\delta^{18}O$	$\delta^{7}Li$
Thin-layer gabbro	HR-10	4.66	4.17
	HR-20	5.16	4.29
	HR-30	4.96	4.78
	HR-40	5.14	4.90
	HR-80	5.20	3.50
Metasediment	0903-6	9.03	0.97
	0903-7	9.42	-0.09
	0903-9	10.3	-0.45
	0903-10	10.8	1.52
	0903-12	13.6	0.66

Table S4. Durations and results of synthetic experiments in a forsterite-water system.

Run number	BE1001	BE1003	BEP1001	BEP1002	BEP1003
Duration (h)	26	110	24	50	110
Starting material§					
[SiO_2]	35.5	35.5	38.9	38.9	38.9
[MgO]	48.1	48.1	52.9	52.9	52.9
[H_2O]	15.9	15.9	7.76	7.76	7.76
[Li]	1088	1088	1064	1064	1064
δ^7Li	13.04	13.04	13.56	13.56	13.56
Weight (mg)	10.8	9.1	11.6	10.7	11.2
Run product					
Olivine†					
[SiO_2]	42.8 (0.2)$^{n=20}$	42.3 (0.1)$^{n=20}$	42.3 (0.5)$^{n=15}$	42.1 (0.6)$^{n=15}$	42.8 (0.5)$^{n=15}$
[MgO]	57.2 (0.2)$^{n=20}$	57.7 (0.1)$^{n=20}$	57.7 (0.5)$^{n=15}$	57.9 (0.6)$^{n=15}$	57.2 (0.5)$^{n=15}$
[Li]	47 (8)$^{n=25}$	43 (5)$^{n=45}$	85 (6)$^{n=77}$	75 (2)$^{n=101}$	38 (2)$^{n=39}$
δ^7Li	–	12.0 (1.5)$^{n=7}$	11.4 (2.6)$^{n=23}$	13.2 (1.8)$^{n=45}$	10.2 (1.7)$^{n=42}$
Fluid‡					
[Li]	–	7496	7920	19258	17005
δ^7Li	–	13.1	13.7	13.6	13.7
$\Delta^7Li_{olivine\text{-}fluid}$	–	-1.1	-2.4	-0.4	-3.5

All the experiments were conducted under conditions of 2 GPa and 900 °C.
Units of elemental abundances are in cg/g, but in µg/g for Li.
§ Elemental and isotope abundances of starting materials were determined by using XRF, and wet chemistry followed by ICP-MS and MC-ICP-MS.
† Elemental and isotope abundances of synthesized olivine were determined by SEM-EDX and SIMS. Variation of the abundances among *n* analyses (1σ) is shown in parenthesis.
‡ Elemental and isotope abundances of Li in fluid that should be retained during experiments were estimated based on mass-balance calculation using the elemental and isotope abundances of Li in the starting material and the synthesized olivine.
– : not available
